# PROMIS, global analysis of PROtein–metabolite interactions using size separation in *Arabidopsis thaliana*

**DOI:** 10.1074/jbc.RA118.003351

**Published:** 2018-05-31

**Authors:** Daniel Veyel, Ewelina M. Sokolowska, Juan C. Moreno, Sylwia Kierszniowska, Justyna Cichon, Izabela Wojciechowska, Marcin Luzarowski, Monika Kosmacz, Jagoda Szlachetko, Michal Gorka, Michaël Méret, Alexander Graf, Etienne H. Meyer, Lothar Willmitzer, Aleksandra Skirycz

**Affiliations:** From the ‡ Department Willmitzer, Max Planck Institute for Molecular Plant Physiology, 14476 Potsdam and; § MetaSysX GmbH, 14476 Potsdam, Germany

**Keywords:** metabolomics, protein–protein interaction, proteomics, systems biology, chromatography, ligand-binding protein, protein–small molecule interactions, size-exclusion chromatography

## Abstract

Small molecules not only represent cellular building blocks and metabolic intermediates, but also regulatory ligands and signaling molecules that interact with proteins. Although these interactions affect cellular metabolism, growth, and development, they have been largely understudied. Herein, we describe a method, which we named PROtein–Metabolite Interactions using Size separation (PROMIS), that allows simultaneous, global analysis of endogenous protein–small molecule and of protein–protein complexes. To this end, a cell-free native lysate from *Arabidopsis thaliana* cell cultures was fractionated by size-exclusion chromatography, followed by quantitative metabolomic and proteomic analyses. Proteins and small molecules showing similar elution behavior, across protein-containing fractions, constituted putative interactors. Applying PROMIS to an *A. thaliana* extract, we ascertained known protein–protein (PPIs) and protein–metabolite (PMIs) interactions and reproduced binding between small-molecule protease inhibitors and their respective proteases. More importantly, we present examples of two experimental strategies that exploit the PROMIS dataset to identify novel PMIs. By looking for similar elution behavior of metabolites and enzymes belonging to the same biochemical pathways, we identified putative feedback and feed-forward regulations in pantothenate biosynthesis and the methionine salvage cycle, respectively. By combining PROMIS with an orthogonal affinity purification approach, we identified an interaction between the dipeptide Tyr–Asp and the glycolytic enzyme glyceraldehyde-3-phosphate dehydrogenase. In summary, we present proof of concept for a powerful experimental tool that enables system-wide analysis of PMIs and PPIs across all biological systems. The dataset obtained here comprises nearly 140 metabolites and 5000 proteins, which can be mined for putative interactors.

## Introduction

Small molecules represent cellular building blocks and metabolic intermediates and also regulatory ligands and signaling molecules, exerting their functions via interaction with macromolecules, most commonly proteins. In fact, one way to see the cell is as “an entity in which proteins are embedded in a sea of metabolites” (1). In line with this, it has been speculated that many more small molecules than known today interact, and by doing so they modulate the function of their protein partners (2, 3).

Until very recently, protein–metabolite interaction (PMI)^3^ studies were hindered by a lack of simple, *in vivo*-like methods for fishing out the interactors. Recent years have seen significant technological advances, allowing for “omics”-scale analysis of PMIs, starting either from a protein or a metabolite of interest that is used to capture the respective metabolite or protein partners from native cellular lysate. Advances in proteomics and metabolomics, increased sensitivity, and better compound identification helped in improving PMI studies. The most promising approaches include the following: 1) purification and characterization of protein–metabolite complexes using affinity-tagged protein baits (4, 5); 2) identification of protein partners using small molecules as affinity baits (6); 3) drug-affinity–responsive target stability assay (DARTS) (7) and thermal proteome profiling (8, 9), both exploiting differences in the stability between unbound and small-molecule–bound proteins to find protein targets of drug compounds; and 4) chemoproteomic methods (10), taking advantage of chemically modified small molecules, which upon binding covalently label their protein partners.

While showing certain success, these methods are limited to the metabolite or protein bait, and thus are not suitable for global analysis of PMIs. We therefore set out to test other approaches that allow a system-wide detection of metabolite–protein complexes and that thereby obviate the need for metabolite or protein bait (11).

Our starting point was the notion that the small-molecule pool of each biological system is distributed between two states: either bound to a protein (subsequently called “bound”) or not bound to a protein (subsequently called “free”). These two forms differ in one main and simple parameter, size. Thus, employing size-separation methods should separate free small molecules in low-molecular weight fraction(s) and metabolite–protein complexes in high-molecular weight fraction(s). We have proven this assumption by demonstrating that the application of a simple size-filtration to a native cellular lysate is sufficient to separate bound from free small molecules (11). Building on this observation, we have additionally shown that size-exclusion chromatography (SEC) can be used to separate small-molecule–protein complexes based on their molecular weight. We detected nearly 100 different polar metabolites co-eluting with the protein fraction and displaying one or several discrete peaks across the chromatographic separation range, indicating the presence of specific protein–small-molecule complexes (11).

Our previous work provided a proof of concept but lacked final experimental confirmation, *i.e.* the identification of known as well as previously unknown small-molecule–protein complexes. Combining SEC separation with parallel metabolomic and proteomic analysis of the fractions using co-elution to indicate interaction, we here demonstrate that SEC is suitable for the identification of protein–small-molecule complexes. This is on par with studies demonstrating SEC applicability for the characterization of protein–protein complexes (12–14). We believe that our approach, which we named PROtein–Metabolite Interactions using Size separation (PROMIS), describes a generic method that allows system-wide detection of protein–small-molecule interactions across different biological systems.

## Results

### Size-exclusion chromatography of soluble plant cell extracts separates protein–small-molecule complexes

**Figure 1.**
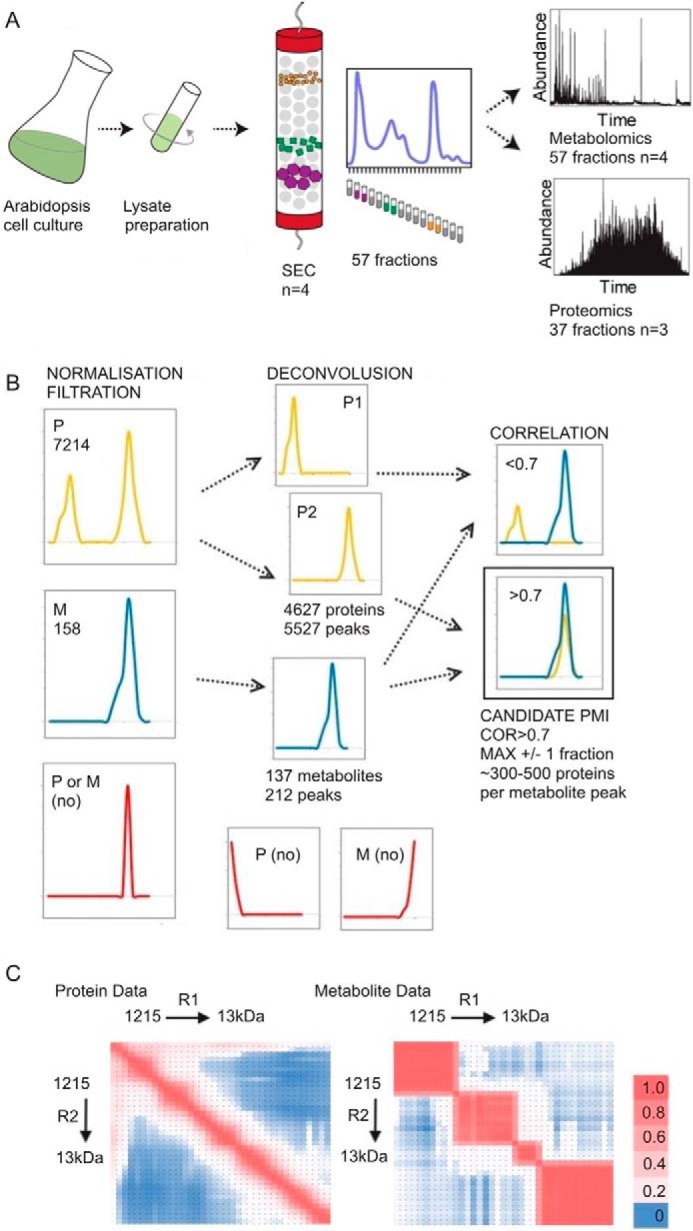
**Experimental workflow.** *A,* schematic representation of the PROMIS experiment. *B,* schematic representation of the data analysis for metabolites and proteins detected in the protein-containing fractions. *P*, protein; *M*, metabolite. In addition, examples of proteins and metabolites filtered out from the final dataset are shown. *C,* data reproducibility calculated using deconvoluted protein and metabolite data. Heat map of Pearson correlation calculated between corresponding fractions between replica 1 and replica 2. Results are comparable with any other comparison, *e.g.* between replica 1 and replica 3.

The goal of this study was to develop a workflow to allow global monitoring of protein–small-molecule complexes. The overall strategy is outlined in Fig. 1, *A* and *B* and in Ref. 11. In brief, a native cellular extract (soluble fraction) was prepared from *Arabidopsis thaliana* cell cultures. Protein and small-molecule complexes were separated by size-exclusion chromatography (SEC).

A total of 57 fractions (A01–D04) collected from four independent biological replicas were subjected to metabolomic analysis, as described previously (11). These fractions comprised 37 protein-containing fractions (A04–C10), referred to as “separation range” (1215 to 13 kDa), and 17 protein-free fractions collected up to one total mobile-phase volume of the column. As expected, the majority of the metabolic features (signal from LC-MS analysis representing specific *m*/*z* and retention time; putative small molecule) were detected in fractions outside the protein separation range, as nonprotein bound. However, 4229 unique metabolic features eluted in the protein-containing fractions, displaying one or several discrete peaks across the separation range, indicating their presence in specific protein complexes (Dataset S1*A*).

In total, 342 metabolic features could be annotated to a metabolite using the exact mass and retention time of a reference-compound library, of which 158 (46%) were present in the protein fractions (Dataset S1*B*). These 158 metabolites included 18 nucleosides and nucleotides, 8 amino acids, 12 cofactors, and 106 dipeptides. Note that the remaining metabolic features correspond to hundreds of additional small molecules, but as we could not assign their identity, we removed these from the analysis.

Next, the same fractions were analyzed for their protein composition using our proteomics platform. Proteins were analyzed in the 37 fractions from three of the four independent biological replicas spanning the entire separation range. In total, we identified 7214 proteins (Dataset S2). Subcellular localization analysis using SUBA 4.0 (15) confirmed that these were mainly of cytoplasmic origin (cytosol, mitochondrion, and plastid), with a significant under-representation of membrane-associated proteins (Fig. S1).

Analysis of the data revealed that in a number of cases there was only one peak for a given metabolite or a given protein and in other cases multiple peaks for one given metabolite or protein. In the case of proteins, this can be easily explained by the presence of the given protein in mono- and oligomeric state and/or in homo- and hetero-oligomers. As for metabolites, several of them, such as co-factors, will have multiple protein partners and thus would be present in several protein complexes, eluting at different times from the size-exclusion column.

As stated above, a first approach of assigning a given metabolite to a potential protein partner is based on the assumption that if present as one complex their chromatographic behavior should be highly similar. To be able to apply this criterion also in case of multiple protein or metabolite peaks, as described above, we split the data profiles into single peaks by finding local valleys in the data; this is referred to as deconvolution.

We then applied three additional filters to the dataset. We first only took into further account proteins and metabolites that were found in all three/four replicas. Second, to exclude spurious signals, we limited the analysis to proteins and metabolites that appeared at least in three consecutive fractions. Third, we removed proteins and metabolites that peaked near the start (1215 kDa) or the end (13 kDa) of the protein separation range, respectively. Applying these three filters plus the deconvolution resulted in a final dataset of 4627 proteins (appearing in 5527 protein peaks) and 137 metabolites (appearing in 212 metabolite peaks) (Dataset S3, *A* and *B,* and Dataset S4). A median calculated from the independent experiments was used to calculate a Pearson correlation between metabolites and proteins to delineate potential interactors (Dataset S5). To define potential interactors, we used a Pearson correlation of >0.7 and allowed deviation of peak maxima of ±1 fraction (COR >0.7 and MAX ± one fraction) (for rationale, see below). By doing so, we found ∼300–500 proteins co-eluting with every metabolite peak, thus representing the potential binding partner.

One important observation was the remarkable reproducibility between the independently performed experiments. The Pearson correlation calculated between corresponding fractions between any two replicas was on average >0.95 for both proteins and metabolites (Fig. 1*C* and Dataset S6, *A* and *B*).

**Figure 2.**
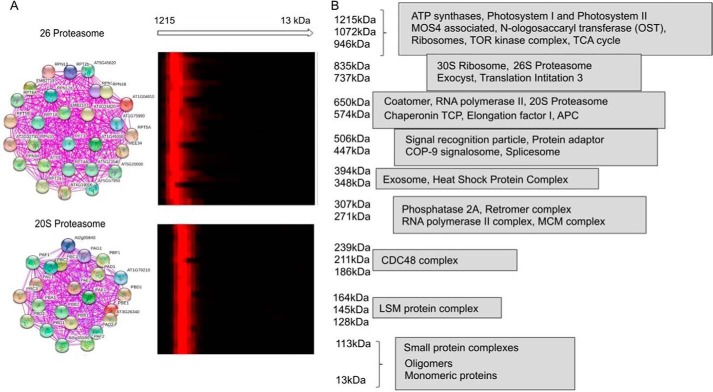
**Presence of known protein–protein complexes confirms the PROMIS approach.** *A,* co-elution of 26S and 20S proteasome subunits (*left panel,* figure generated by Stitch) presented as a heat map. Protein abundance was normalized to the maximal intensity measured across the separation range. *Red,* presence; *black,* absence. *B,* identity of the known protein–protein complexes across PROMIS separation range inferred by querying Stitch database with lists of co-eluting proteins. Note, for complexes of >960 kDa, see Table S2.

Finally, we inspected the quality of our experimental dataset by checking the co-elution of the proteasome subunits, as done previously (12). The 26S proteasome holoenzyme consists of two regulatory particles (RPs) capping each end of the barrel-shaped catalytic 20S core particle (CP) (16, 17). CP and RPs are composed of multiple subunits, all sized roughly 25–30 kDa, and eluted as two separate complexes. In our experiment we identified 23 and 29 CP and RP subunits, respectively, of which 23 and 27 co-fractionated together (COR >0.9), with the maximum intensity measured in the two neighboring fractions corresponding to complexes of 574/650 kDa and 735/835 kDa (Fig. 2*A*). Proteasome co-elution demonstrated that allowing deviation of peak maxima by ±1 adjacent fraction is a rationale criterion to define potential interactors. Importantly, the proteasome is just one of the multiprotein complexes present in the PROMIS dataset (Fig. 2*B*). In this study, we focused on the PMIs. However, our dataset can be used to query protein–protein complexes on par with the study of Ref. 12.

In summary, we generated a highly reproducible dataset comprising roughly 140 annotated metabolites and 5000 proteins, which can be used to investigate protein–protein and protein–small-molecule complexes.

### Confirmation of the PROMIS approach using known protein–small-molecule interactions

**Figure 3.**
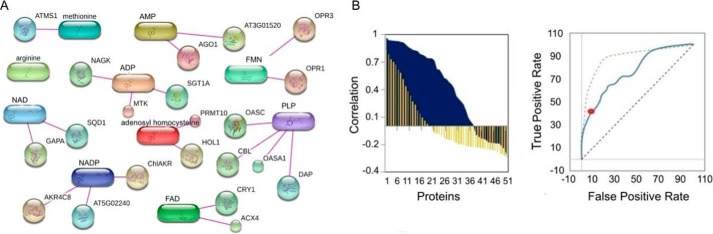
**Presence of known protein–protein and protein–metabolite complexes confirms the PROMIS approach.** *A,* 21 of the 51 known protein–metabolite interactions that passed >0.7 Pearson correlation cutoff in the PROMIS experiment. *B*, distribution of the Pearson correlation measured for the 51 known interactions (*dark blue*) *versus* 51 random interactions (*yellow bars*, average *n* = 10) selected from the PROMIS experiment (*left panel*) was used to calculate the ROC curve (*right panel*). The *red dot* indicates the ratio of true positives to false positives using a >0.7 Pearson correlation cutoff.

The data described above demonstrate that our experimental procedure retains the integrity of protein–protein complexes. We next aimed to confirm the presence of small-molecule–protein interaction partners by querying the data for the presence of known small-molecule–protein complexes as defined by the co-elution criterion. To this end we used the Stitch database (18) to identify known protein–metabolite interactions, however, restricting our analysis to cases that were experimentally proven in *Arabidopsis*. Moreover, we restricted the analysis to cases where both metabolite and protein were detected anywhere in our dataset. This resulted in 51 interactions reported for 48 proteins and 13 metabolites detected in our PROMIS experiment (Table S1). We subsequently asked whether or not, by applying the criterion of co-elution, we would retrieve a significant number of the metabolite–protein complexes from the results of the PROMIS experiment. Indeed, this was clearly the case. Of the 51 interactions we could have potentially found in our dataset, 21 interactions were retrieved when applying a Pearson correlation cutoff of 0.7 between protein and metabolite elution (Fig. 3*A*). This is five times more than expected and thus is statistically significant (Fisher exact test <0.05), greatly supporting our experimental approach (Fig. 3*B*). Examples include interaction between enzymes involved in oxylipin metabolism, 12-oxophytodienoate reductase 1 (OPR1) and OPR3, and the cofactor flavin mononucleotide (FMN) (19), and between amino acid biosynthetic enzymes, cysteine synthase 1 (OASA1) (20) and diaminopimelate aminotransferase (21), and the cofactor pyridoxal phosphate (PLP).

To see how a shift in the Pearson correlation influences the detection of true (known) interactions, we performed an analysis where we varied the Pearson correlation between 1 and −1 (Table S1). As shown in Fig. 3*B*, lowering the correlation cutoff drastically reduced the true positives to false positives ratios, leading us to decide against it. Based on these results, we decided to apply a Pearson correlation value of 0.7 for the analysis of the entire dataset. The same result is also reflected in the receiver operating characteristic (ROC) curve.

Finally, we were interested in finding out whether or not there is a relation between binding affinity and our results. To this end, we tried to gather *K_d_* data for the 51 known interactions. However, only for six examples are *K_d_* values reported, ranging from low millimolar to mid nanomolar. Obviously, this dataset is too small to allow any further conclusion with respect to the applicability of our experimental approach to specific (high-affinity) binding events, for example.

### Co-elution supports the existence of a number of protein–cofactor interactions previously not demonstrated in A. thaliana

In the previous paragraph we asked whether the co-elution criterion can be used to retrieve proven metabolite–protein interactions. As the Stitch database contains a comprehensive listing of essentially all proven metabolite–protein interactions, we next wondered whether the SEC data would allow gaining additional support for metabolite–protein interactions validated in systems other than *A. thaliana* based on protein homology. To this end, we decided to test eight cofactors present in our metabolomics dataset (Table S2).

**Figure 4.**
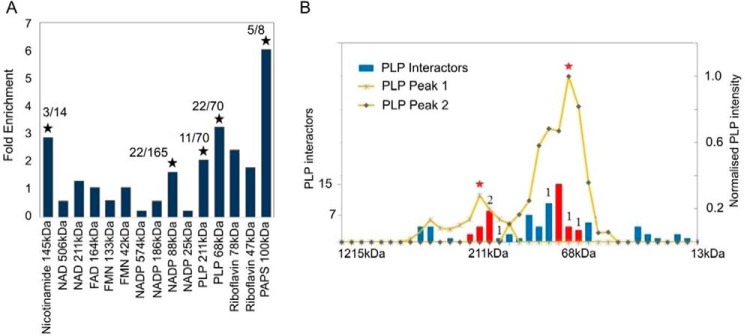
**PROMIS can be used to confirm predicted protein–metabolite interactions.** *A* enrichment of predicted protein partners for the eight cofactors present in the SEC experiment, separately for each elution peak. *Star* indicates significance (Fisher exact test <0.05). Also given is the number of proteins found as interactors in the PROMIS dataset *versus* the total number of Stitch-predicted interactors. *B,* overlay of the PLP elution profile, normalized to the maximum intensity measured across the separation range, and the number of PLP interactors eluting in different fractions (based on the elution maxima of the protein peaks). The number indicates interactions confirmed for the *Arabidopsis* proteins. *Red bars* represent fractions around the two major elution maxima.

PLP (22) will be described here as an example. Of the 138 proteins annotated in the Stitch database as interacting with PLP, we found 76 in our proteomics data of the PROMIS experiment (Table S3). For only six of these 76 proteins, the interaction with PLP had been demonstrated previously for *A. thaliana*. For the remaining 70 proteins, whose interaction with PLP had been shown in systems other than *A. thaliana,* we used the most homologous proteins for the subsequent analysis. In our dataset, PLP eluted as two distinct peaks with maxima at 211 and 68 kDa. The list of proteins predicted as potential interactors (COR >0.7 and MAX ±1 fraction) totaled 355 and 414 proteins for the two peaks, respectively. Of the 70 proteins annotated as PLP binding in the Stitch database, 11 and 20 were contained in the first and second peak, respectively, which is 2.1 and 3.2 times higher than expected by chance (*F* test < 0.05) (Fig. 4*A*). Even more striking, when we overlaid the elution profiles of PLP and the known and predicted PLP interactors (76 proteins), we found that 69 of the 76 proteins were contained in the PLP-containing fractions (Fig. 4*B*). The fact that only half of the proteins were detected when applying the cutoff and correlation approach as described above is due to the relative broadness of the PLP elution peaks, with additional local maxima (394 and 88 kDa), albeit too minor to be reliably selected during deconvolution.

In case of the other seven cofactors: for three of them (NADP, adenosine 5′-phosphosulfate, and nicotinamide), the overlaps between PROMIS-predicted interactions and the data from the Stitch database were again significantly higher than expected by chance (Fig. 4*A* and Table S2). For the remaining four, we did not find significant enrichment. In contrast to positive results, as in the case of PLP, negative results are harder to interpret and can have a number of reasons as follows: binding predictions retrieved from Stitch may be false; predictions are correct but the binding is development- and/or environment-specific and thus absent in the cell cultures; or the predictions are correct but the binding is not retained during our experimental procedure.

In summary, SEC can be used to support predicted PMIs. PLP is an example of a metabolite that interacts with a large number of proteins, with a complex elution behavior, which may require adjusting the criteria by which protein interactors are selected. Still, it is also an exciting example, as the data described above clearly demonstrate that a large number of protein–metabolite complexes are retained during our experimental procedure, which was not anticipated before (see “Discussion”).

### PROMIS can be used to trace protein partners of exogenously added small molecules

**Figure 5.**
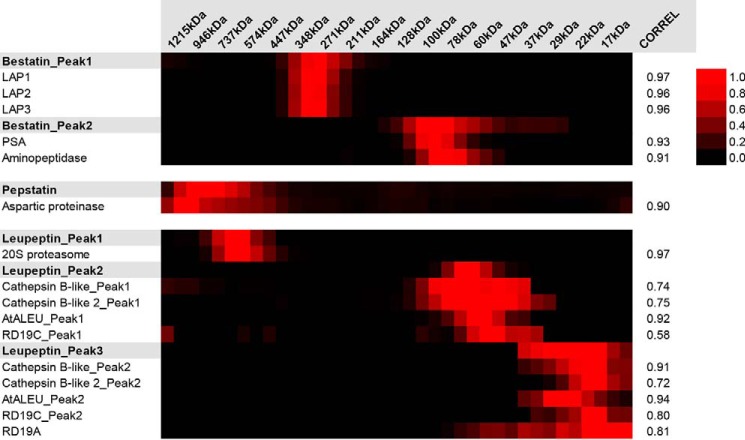
**Proteinase inhibitors co-elute with their respective protease targets in the PROMIS experiment.** Shown is a heat map of the elution profiles measured for proteinase inhibitors and their protease targets. Data were normalized to the maximum intensity of given small molecule or protein measured across the separation range. Also given is the Pearson correlation.

To further challenge our approach, we tested whether PROMIS can be used to reproduce known PMIs. Our attention was drawn to the commercial protease inhibitors present in the lysis buffer. If PROMIS is effective in separating true protein–metabolite complexes, we expected to find these in the protein-containing fractions, co-eluting together with their protease targets. Both of the above assumptions proved true. Three of the six commercial protease inhibitors present in the lysis buffer and known to exhibit reversible binding behavior separated in the protein-containing fractions in minimum one and maximum three distinctive elution peaks (Fig. 5; Dataset S7). Remarkably, in each case the elution behavior of the protease inhibitor could be traced down to a known protease target.

More specifically, bestatin is an aminopeptidase inhibitor. Of the five aminopeptidases present in our dataset and annotated in Stich as bestatin interactors, all five, including the family of the three leucine aminopeptidases (LAP1–3), eluted together with either one of the two bestatin peaks. Pepstatin A, an aspartyl protease inhibitor, eluted as one peak mirrored by the elution behavior of one of the four aspartyl proteases present in the PROMIS dataset and annotated in Stich as pepstatin interactors. Leupeptin, a cysteine and serine protease inhibitor, had three distinct elution peaks. Peak 1 coincided with the elution behavior of the 20S proteasome complex (23). Peaks 2 and 3 can be explained by the elution behavior of the cysteine proteases. Strikingly, of the seven cysteine proteases found in our dataset, four had two elution peaks, and for all four, the two peaks co-eluted with the two peaks of leupeptin (peak 2 and peak 3). These were two cathepsin-like proteases, thiol protease aleurain, and RD19C. One additional cysteine protease, RD19A, co-eluted with peak 3 of leupeptin. In conclusion, PROMIS was successful in separating small-molecule protease inhibitors with their respective proteases. In this way we further validate our approach, but also demonstrate that PROMIS can be considered as a method for finding protein interactors of not only endogenous ligands (metabolites), but also of drugs and agrochemicals.

### Putative regulatory mechanisms revealed by co-elution behavior during PROMIS

As described above, applying correlation between elution behavior of proteins and metabolites as one criterion to identify potential candidates for protein–metabolite complexes is helpful and enriches in a statistically significant manner for known protein–metabolite pairs. However, as a rule it does not allow us to identify the single protein–metabolite pair. In consequence, either a number of PROMIS experiments have to be performed with varying elution characteristics, thus allowing narrowing down the potential partners, or orthogonal approaches need to be used. In the following, we describe the successful application of one of these orthogonal approaches. We introduced biochemical knowledge into the query to narrow down potential protein candidates for a given metabolite. To this end we decided to query the dataset for co-elution of metabolites with proteins belonging to the same biochemical pathway. The underlying reasoning is that for many biochemical pathways it is known that metabolites from within the pathway exert a regulatory function on another enzyme of the same pathway.

Following this notion, we identified all cases in our dataset where proteins and metabolites of one given biochemical pathway fulfill the criterion of co-elution using the plant metabolic pathway database as reference. Two examples will be discussed in more detail below, pantothenic acid and methylthioadenosine.

**Figure 6.**
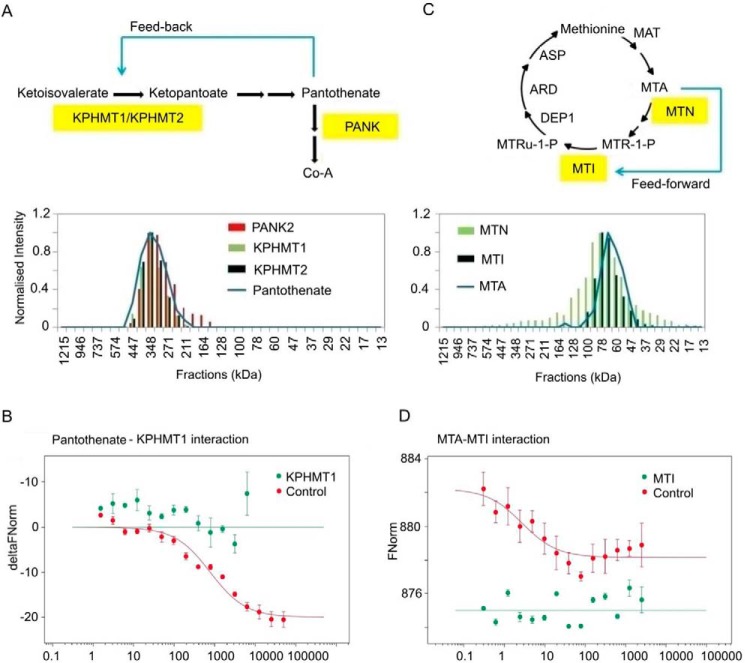
**Putative feedback regulatory mechanism disclosed by the PROMIS analysis.** *A,* schematic overview of pantothenate synthesis in plants (*upper panel*) (24). Enzymes highlighted in *yellow* co-eluted with pantothenate in the SEC experiment (*lower panel*). *Blue* indicates a putative feedback regulation. *B,* MST measurements testing the interaction between KPHMT1 and pantothenate. Data are presented as difference in normalized fluorescence (Δ*FNorm*) calculated between bound and nonbound KPHMT1. Data are mean ± S.D., *n* = 3. His_6_ peptide was used as negative control. *C,* schematic overview of the methionine salvage pathway in plants (*upper panel*) (29). Enzymes highlighted in *yellow* co-eluted with MTA in the SEC experiment (*lower panel*). *Blue* indicates a putative novel feed-forward regulation. *D*, MST measurements testing the interaction between MTI and MTA. Data are presented as normalized fluorescence (*FNorm*). Data are mean ± S.D., *n* = 3–5 (technical replicates). His_6_ peptide was used as negative control.

Pantothenic acid (pantothenate, vitamin B_5_) is a precursor of the important co-factor coenzyme A (CoA). The pantothenate pathway is best described in *Escherichia coli* and was used as a blueprint to elucidate pantothenate metabolism in plants (24). Two *panB* genes encoding ketopantoate hydroxymethyltransferase (KPHMT) 1 and 2, a single *panC* gene encoding pantothenate synthetase (PS), and two *panK* genes encoding pantothenate kinase (PANK) 1 and 2 were reported in *Arabidopsis* (Fig. 6*A*). In the SEC experiment, pantothenic acid elutes as a single, sharp peak with the maximum at 348 kDa (Fig. 6*A*). Not surprisingly, but reassuringly, PANK2 is one of the 434 proteins co-eluting with pantothenate. PANK2, which catalyzes the first step of CoA synthesis, uses pantothenate as a substrate, which is likely the reason for the co-elution behavior (25). Two more enzymes of the pantothenate pathway, KPHMT1 and KPHMT2, catalyzing the first committed step of pantothenate biosynthesis, upstream of PS (Fig. 6*A*), also display co-elution behavior with pantothenic acid (24). We next decided to test directly whether pantothenate binds to either KPHMT. To this end, we produced recombinant KPHMT1 from *A. thaliana* in *E. coli* and tested the purified protein for its binding to pantothenate using microscale thermophoresis (MST). MST is a fairly new biophysical analysis tool for analyzing interactions between proteins and metabolites, building on the principle that microscopic temperature gradients lead to movement of biological molecules (26). Changes in size, conformation, charge, and/or hydration shell, which are likely to occur during complex formation, alter the movement and are thus indicative of a binding event. Using this approach, we could demonstrate that indeed pantothenate binds KPHMT1 with a *K_d_* of 500 μm (Fig. 6*B*), suggesting its involvement in feedback regulation controlling the rate of pantothenate synthesis. Importantly, this would be a conserved mechanism as PanB, a bacterial homolog of KPHMT1, was shown to be subjected to the allosteric pantothenate inhibition in concentrations above 500 μm (27).

Methylthioadenosine (MTA) is a naturally occurring sulfur-containing nucleoside, a by-product of ethylene, polyamine, and nicotinamide synthesis. MTA is rapidly metabolized in the cell by the activity of 5′-methylthioadenosine nucleosidase (28). The product of this reaction, 5′-methylthioribose (MTR), is further recycled into methionine in the so-called methionine salvage pathway. In the SEC experiment MTA elutes in two peaks with maxima at 835 and 68 kDa (Fig. 6*C*). Again, not unexpectedly, we found MTN1 among the 444 proteins co-eluting with MTA (68 kDa peak). More interestingly, MTA also co-fractionated with methylthioribose-1-phosphate isomerase (MTI) (29, 30), catalyzing a downstream reaction of the methionine salvage pathway in which 5′-methylthioribose-1-phosphate (MTR-1-P) is converted into 5′-methylthioribulose-1-phosphate (MTRu-1-P) (Fig. 6*C*). Again, using recombinant *Arabidopsis* MTI protein and MST, we could demonstrate MTI–MTA interaction with *K_d_* of 4 μm (Fig. 6*D*), suggesting possible regulation, a putative feed-forward loop, controlling the rate of the methionine salvage pathway. MTA was shown previously to act as an allosteric inhibitor in the ethylene, polyamine, and nicotinamide synthesis pathways (31), which release MTA as a by-product, but its regulatory function in the methionine salvage pathway has not been described so far.

In summary, we could demonstrate that querying co-elution of small molecules and enzymes from the same metabolic pathway is an efficient way to look for new regulatory mechanisms.

### Novel interaction between dipeptide Tyr–Asp and glycolytic enzyme glyceraldehyde-3-P dehydrogenase (GAPC) revealed by combination of PROMIS and affinity purification (AP)

The pantothenate–KPHMT1 and MTA–MTI interactions were selected using a combination of PROMIS together with existing biochemical knowledge. However, for many metabolites this would be impossible because there is no sufficient information regarding either their metabolism or biological function. Diverse dipeptides, which we see present in protein-containing fractions, are just one example of such small molecules. In such instances, PROMIS needs to be supported with an orthogonal experimental method.

**Figure 7.**
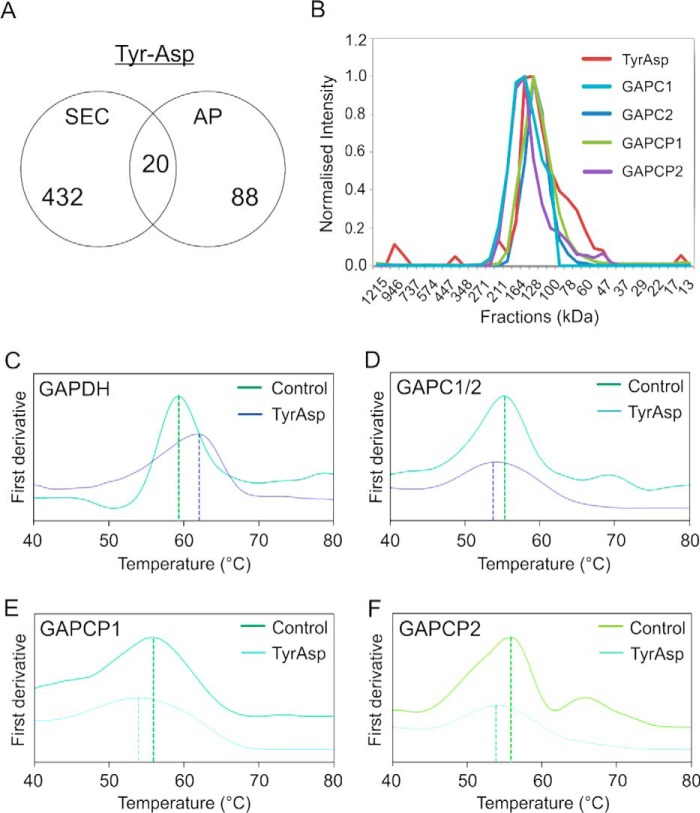
**Identification and validation of a novel protein–metabolite interaction by means of PROMIS, AP, and nanoDSF.** *A,* Venn diagrams comparing putative Tyr–Asp interactors derived from PROMIS and AP experiments. *B,* elution pattern of Tyr–Asp and the GAPC family enzymes in the PROMIS dataset. *C,* nano-DSF analysis of the melting profile of GAPDH protein with and without Tyr–Asp (500 μm). *D,* nano-DSF analysis of the melting profile of GAPC1/C2 protein with and without Tyr–Asp (25 μm). *E,* nano-DSF analysis of the melting profile of GAPCP1 protein with and without Tyr–Asp (25 μm). *F,* nano-DSF analysis of the melting profile of GAPCP2 protein with and without Tyr–Asp (50 μm). Protein concentration of 0.2–0.25 mg/ml was used for the nano-DSF experiments. For the binding assays (*C–F*) one representative replicate of the unfolding curve (with and without Tyr–Asp) was selected (*T_m_* indicated with the *vertical line* was calculated using default settings in the Prometheus NT.48 software); for the complete data (*n* = 4; independent experiments) see Table S5.

Based on its reproducible and specific elution profile, we selected a dipeptide, Tyr–Asp, to test a combination of PROMIS with an AP approach. For this purpose, we used agarose beads coupled to Tyr–Asp either via the NH_2_ group of tyrosine or the COOH group of aspartic acid. After incubation of the beads with total soluble-protein lysate, referred to as input, unspecific binders were removed by washings with tyrosine and aspartic acid, followed by specific elution with Tyr–Asp. The eluate was analyzed by LC-MS (LC-MS/MS) proteomics. A total of 108 proteins were reproducibly identified in the eluate coming from the N′ and C′ beads, constituting putative Tyr–Asp binders (Fig. 7*A*; Dataset 8). In comparison and based on co-elution, PROMIS identified 452 putative Tyr–Asp interactors. Testing either 452 or 108 proteins for Tyr–Asp binding would be unrealistic. However, when we compared PROMIS and AP results, we found an overlap of 20 proteins (Fig. 7*A*; Table S4). Among these proteins, cytosolic glyceraldehyde-3-phosphate dehydrogenase (GAPC1) stood out as its elution profile almost perfectly mirrored the elution profile of Tyr–Asp (Fig. 7*B*). This pointed to a strong association between Tyr–Asp and the GAPC1 protein. Notably, other members of the GAPC family (GAPC2, GAPCP1, and GAPCP2) also co-fractionated with Tyr–Asp as shown in Fig. 7*B*, suggesting a shared binding specificity for the Tyr–Asp dipeptide.

To validate the binding, we first tested a commercially available human homolog of GAPC1, GAPDH. Subsequently, plant GAPC1/2, GAPCP1, and GAPCP2 were overexpressed and purified from *E. coli*. Binding was probed using nano-DSF technology from Nanotemper (Prometheus NT.48; see under “Experimental procedures”). Prometheus NT.48 traces protein thermal stability by recording changes in the tryptophan residues during protein unfolding in an increasing temperature gradient. The melting temperature (*T_m_*) of GAPDH was calculated as 60 °C, GAPC1/2 to 54 °C, GAPCP1 to 55 °C, and GAPCP2 to 55 °C (Fig. 7, *C–F*; Tables S5 and S6). As expected, addition of the substrate, glyceraldehyde 3-phosphate (3PGA), significantly shifted the *T_m_* of all tested proteins, which is indicative of binding (Table S5). Analogous results were obtained for Tyr–Asp. More specifically, 500 μm Tyr–Asp stabilized GAPDH by ∼1.1 °C, whereas 25–50 μm Tyr–Asp destabilized GAPC1/2, GAPCP1, and GAPCP2 by ∼1, 2, and 1.5 °C, respectively (Fig. 7, *C–F*; Tables S5 and S6). The obtained results validate the interaction between Tyr–Asp and plant glyceraldehyde-3-P dehydrogenases. The interaction is conserved in the human enzyme.

Finally, to explore the specificity of the binding, several dipeptides (Gly–Pro, Pro–Glu, Leu–Phe, Thr–Met, His–Tyr, and Tyr–Leu) and single amino acids (Tyr and Asp) were tested. Although no binding was measured for plant glyceraldehyde-3-P dehydrogenases, the *T_m_* of the human GAPDH was affected by the presence of two other Tyr-containing dipeptides, His–Tyr and Tyr–Leu (Table S6).

In summary, by combining PROMIS and AP, we revealed a novel interaction between dipeptide Tyr–Asp and a family of *Arabidopsis* glyceraldehyde-3-P dehydrogenases. Importantly, Tyr–Asp binding is also conserved for human GAPC. Glyceraldehyde-3-P dehydrogenases are glycolytic enzymes but have been also implicated in transcriptional regulation, signal transduction cascades, DNA repair, and apoptosis (so-called moonlight functions) (32). Considering recent reports pointing to the regulatory functions of dipeptides (33), it will be extremely interesting to investigate physiological consequences of the Tyr–Asp–glyceraldehyde-3-P dehydrogenase interaction.

## Discussion

Most common strategies to elucidate protein–small-molecule interactions start with a single protein or metabolite bait and exploit affinity purification in combination with MS detection (5, 6). These methods are labor-intensive, often require transgenic lines, and provide information restricted to the used bait. In consequence, although recent literature suggests a still unexplored wealth of protein–metabolite interactions, their true extent remains unknown (1).

As described here, using co-elution behavior during SEC offers an attractive means to obtain a much more comprehensive view of potential protein–metabolite complexes. SEC is an accepted method to separate and characterize protein–protein complexes. Herein, and to our knowledge for the first time, we report its suitability for the global identification of protein–small-molecule interactions. We could previously demonstrate that small molecules are retained in the protein complexes during lysate preparation and size separation, without the need for prior chemical cross-linking (11). This approach seems to be surprisingly robust given the time it takes to complete the separation (∼3 h), the various dilutions introduced both during lysate preparation and subsequent fractionation step, and the largely polar nature of the small molecules that can easily dissolve in the used buffer. This would indicate that many of the cellular PMIs are in fact very stable, possibly due to a combination of low *K_d_* values and long dissociation time. However, this seems to be in contrast with the *K_d_* value of 500 μm measured for the pantothenate–KPHMT1 interaction. Therefore, one has to assume that additional factors also may contribute to the observed stability that is not reflected when determining *K_d_* values in a binary system. Such factors may be additional protein partners, post-translational modifications, or even co-eluting RNAs.

As PROMIS is a novel approach, we were careful in performing a number of positive and negative controls that will be summarized here. 1) Already in our preceding study, protein-free small-molecule extract was used as a negative control to exclude the unlikely possibility that free metabolites would elute in the high-molecular weight fractions. Indeed, this was not the case (11). 2) As described under “Results,” when querying how many of the known protein–metabolite interactions would be represented in our data, we showed that our approach retrieves five times more of the known PMIs than expected by chance. 3) We could separate exogenously added proteinase inhibitors together with their respective protease targets.

In a follow-up experiment, we could also demonstrate that PROMIS can be used to discover novel protein–small molecule interactions. 1) By combining the co-elution data with biochemical knowledge, we were able to predict two novel PMIs, namely KPHMT1–pantothenate and MTI–MTA, for which we could trace down the binding and determine the *K_d_* value of the binary complex. 2) By combining PROMIS with AP, we identified a novel interaction between Tyr–Asp and the glyceraldehyde-3-P dehydrogenase enzyme. It is worth mentioning that an analogous combination of PROMIS and AP experiments led to the discovery of a novel interaction between 2′,3′-cAMP small molecule and Rbp47b protein (34). Importantly, we could show that the 2′,3′-cAMP binding to the Rbp47b is relevant *in vivo*-promoting stress granule formation.

Based on the above evidence, we are convinced that our method, named PROMIS, provides a reliable means to isolate, separate, and characterize protein–metabolite interactions from native cellular lysate, close to the *in vivo* situation. Most importantly and in contrast to other methods, a single PROMIS experiment can be used to deduce interactions between many metabolites and many proteins, enabling a system-wide view into the interactome. Notably, both protein–protein and protein–small-molecule complexes can be traced in a single experiment. The PROMIS method is generic and can be used in any organism and sample type without the need for transgenic lines, as is the case for the tandem affinity approaches (5). In contrast to DARTS (7) and cellular thermal shift assay/thermal proteome profiling (TPP) (35), PROMIS operates in near-cellular metabolite concentrations and does not require small-molecule modifications such as attachment to agarose beads (6). As such, PROMIS is less likely to generate false positives related to a high concentration of either protein or metabolite bait, as well as false negatives related to small-molecule modifications.

On the down side, PROMIS is in its nature poorly predictive, as co-elution is an indication, and not evidence for interaction. Predictive power is related to the resolution, which depends on the separation range of the column, number of collected fractions, and sensitivity of the proteomic and metabolomic platform. In an experiment like ours, every metabolite is correlated with several hundred proteins, of which merely a handful constitute true binders. As with any other omics study that relies on correlation to define associations, a larger dataset comprising multiple PROMIS experiments covering developmental, environmental, and/or genetic diversity, will improve predictive power. Nevertheless, even a single experiment such as the one provided here allows meaningful hypothesis without the need for additional experiments. Looking at the co-elution of a metabolite with the enzymes involved in its metabolism proved successful to find novel putative regulatory mechanisms.

As presented herein and using Tyr–Asp as an example, when combined with orthogonal experimental approaches, PROMIS allows us to trace protein interactors also in the absence of literature knowledge. A point of criticism may be that when deciding for a targeted approach such as AP or TPP anyway, PROMIS is needless. But as evident from the above, had it not been for the PROMIS experiment, we would never have selected Tyr–Asp for further analysis. In other words, a PROMIS experiment delineates a set of small molecules that are retained in the protein complexes and thus are (i) likely involved in regulation and signaling, and (ii) are accessible to biochemical characterization. Moreover, as described previously, both AP and TPP, however successful in retrieving true targets, are also known to generate numerous false positives. A combination of the methods (AP, TPP, and PROMIS) can be seen as a logical way to distinguish true from false interactors.

As any other approach that starts with a cellular lysate, there are other points to be considered. The choice of buffering conditions will affect measured interactions with higher salt concentrations favoring hydrophobic over ionic bindings. Many weak and transient interactions may be lost. Crushing organelles may lead to formation of false interactions, which do not occur *in vivo*. Using isolated organelles may circumvent the last issue, but as with the other mentioned approaches that look into protein–protein interaction and PMI, it is rarely done as it is laborious, time-consuming, and often simply infeasible. As a much simpler alternative, we propose to filter the interaction data, taking advantage of the subcellular localization available for a majority of the *Arabidopsis* proteins (15) but increasingly for the small molecules (36). Finally, a modification of the protocol would be required to tap into membrane proteins. For instance, it was demonstrated (8) that inclusion of the mild detergent during cell extraction liberates membrane proteins without affecting protein–ligand interactions, as determined in the TPP study.

One last issue is data analysis. Following the approach taken for protein–protein complexes, we applied deconvolution to split elution profiles into single peaks. This was necessary as we expected that a single metabolite can interact with different proteins, resulting in multiple elution maxima across the separation range. We anticipated that the majority of the small molecules, *e.g.* dipeptides or cyclic nucleotides, will have few specific protein partners, which would justify using Pearson correlation cutoff and peak maxima to define candidate interactors. A single protein peak is expected to correspond to a single metabolite peak, and thus they should mirror each other with respect to their elution pattern. The choice of Pearson correlation of >0.7 was based on the ROC curve, and it seemed to be a good compromise between true positives and the false positives ratio.

Nevertheless, there are metabolites for which multiple proteins would contribute to a single elution peak, obscuring elution profile and data analysis. The extreme case presented here is PLP, which is a cofactor for more than a 140 different enzymatic reactions (22). PLP's elution profile spans 21 of the 37 protein fractions (from 506 to 42 kDa), with two major elution peaks, but also with additional minor maxima that were not selected during deconvolution. If we consider only the major peaks and use our prediction cutoff (COR > 0.7 and MAX ±1 fraction), we retrieve 34 of the 76 experimental protein interactors found in the Stitch database and present in our dataset. But if we consider the whole elution span, we would increase the identification to 69 proteins, which is nearly 90%. Therefore, in cases of metabolites like PLP, co-presence may be a better criterion to define interaction.

In conclusion, PROMIS, based on the co-elution of proteins and metabolites, offers a novel, powerful tool to explore the protein–metabolite interactome, and it could prove essential to understand how differences in the small-molecule interactome contribute to the developmental, environmental, and/or genetic readouts.

## Experimental procedures

All chemicals were acquired from Sigma unless otherwise noted.

### Plant cell cultures

Cell suspension culture PSB-L of *A. thaliana* (L.) Heynh. ecotype *Landsberg erecta*, derived from MM2d cells (38) was grown in MSMO medium, in a continuous photoperiod, at 21 °C, on orbital shaker (110–120 rpm). MSMO medium consists of 4.43 g/liter Murashige and Skoog basal salts with minimal organics (Sigma), 30 g/liter sucrose, 0.5 mg/liter of 1-naphthaleneacetic acid, 0.05 mg/liter of kinetin, pH 5.7, adjusted with 1 m KOH. Cells were harvested at the logarithmic growth phase (7 days after last passage) by filtration and immediately frozen in liquid nitrogen. Experiments were conducted using four independently inoculated, grown, harvested, and extracted cultures.

### Native Arabidopsis lysate preparation

Plant material was collected as described above and pulverized using a liquid nitrogen mortar, and pestle. 1 ml of a lysis buffer was added per 1g of material (25 mm Tris-HCl, pH 7.5, 0.5 m NaCl, 15 mm MgCl_2_, 0.5 mm DTT, 1 mm NaF, 1 mm Na_3_VO_4_, 1× protease inhibitor Mixture, Sigma catalog no. P9599, Steinheim, Germany). Cellular debris was removed by a 10-min centrifugation at 4000 rpm (4 °C). The crude lysate was then subjected to a 1-h ultracentrifugation at 35,000 rpm (4 °C) to obtain a soluble fraction referred to as the native *Arabidopsis* lysate.

### Size-exclusion chromatography

Size-exclusion chromatography was performed at 4 °C as described previously (11). 2.5 ml of soluble fraction corresponding to 50 mg of protein, as determined by Bradford assay, were used for the separations. SEC was performed with a HiLoad 16/600 Superdex 200 preparation grade column (GE Healthcare Life Science, Little Chalfont, UK) connected to an ÄKTA Explorer 10 (GE Healthcare Life Science) operating at 4 °C. The flow rate was set to 0.8 ml/min. 57 fractions of 1.5 ml were collected from a 40- to 125.5-ml elution volume of which 1 ml was dried in a speed vac overnight and stored at −80 °C for metabolomic analysis.

### Extraction and LC-MS analysis of small molecules

Metabolites were extracted from SEC fractions as described previously (11). In short, the collected fractions were extracted using methyl *tert*-butyl ether/methanol/water solvent system to separate proteins, lipids, and polar compounds into pellet, organic, and aqueous phase, respectively (39). After extraction, the aqueous phase was dried in a speed vac and stored at −80 °C until LC/MS analysis. Samples were measured using ultra-performance LC coupled to an Exactive mass spectrometer (ThermoFisher Scientific) in positive and negative ionization mode as described previously (39).

### LC-MS/MS of proteins

Protein concentration of SEC fractions was determined by the Bradford assay (Carl Roth GmbH + Co. KG, Karlsruhe, Germany). An equivalent of 75 μg of protein (if less protein was available, a maximum of 300 μl was used) from fractions A04 to C10 was precipitated in 80% acetone at −20 °C overnight. After pelleting the proteins by centrifugation (4 °C, 20 min, 20,000 × *g*), pellets were resuspended in 18 μl of urea buffer (6 m urea, 2 m thiourea in 40 mm ammonium bicarbonate). Cysteine reduction (using DTT) and alkylation (using iodoacetamide) followed by enzymatic digest using LysC/trypsin mix (Promega Corp., Fitchburg, WI) was done following the instruction manual. Peptide samples were desalted on C18 Empore® extraction discs (3M, Maplewood, MN) STAGE tips using 32.5 μg of digest, and the eluted peptides were concentrated in a speed vac to ∼2 μl and stored at −80 °C until measurement. Dried peptides were resuspended at a concentration of 0.33 μg/μl in 3% acetonitrile, 0.1% TFA. 2 μg were analyzed on an Easy nLC-1000 connected to a Q-Exactive Plus mass spectrometer (both ThermoFisher Scientific Inc.). Peptide samples were separated on a reversed phase Acclaim® PepMap column (C18, 2 μm, 100 Å, 75 μm inner diameter × 150 mm) using Buffer A (0.1% formic acid) and Buffer B (60% acetonitrile, 0.1% formic acid) at a flow rate of 300 nl/min. The gradient started from 3% acetonitrile increasing to 18% over 60 min and further to 30% after 90 min followed by a washout at 60% acetonitrile for 10 min and re-equilibration with 6 μl of Buffer A. The Q-Exactive Plus was interfaced with a Nanospray Flex^TM^ ion source (ThermoFisher Scientific Inc.) with a spray voltage of +2.1 kV, capillary temperature set to 275 °C, and S-lens to *RF* level of 50. We used a data-dependent top-N method that fragmented the top 15 most intense ions per full scan. Full scans were acquired at a resolution of 70,000 with an AGC target 3e6, maximum injection time 100 ms, scan range 300 to 1600 *m*/*z* in profile mode. Each dd-MS2 scan was recorded in profile mode at a resolution of 17,500 with an AGC target of 1e5, maximum injection time 100 ms, isolation window 1.6 *m*/*z*, normalized collision energy 25, and an underfill ratio of 20%. Charges below 2 and above 4 were excluded; the peptide match was set to preferred, apex trigger and exclude isotopes were set to on, and the dynamic exclusion lasted for 15 s.

### Data pre-processing: LC-MS metabolite data

LC-MS metabolite raw data were analyzed using Refiner MS 9.0.4 (Genedata AG, Basel, Switzerland) using the following activities and settings: removal of chemical noise (chromatogram smoothing three scans, estimator moving average, RT window 51 scans, quantile 50%, intensity threshold for clipping 750, RT and *m*/*z* structure removal enabled with a minimum RT length of 5 scans, and a minimum *m*/*z* length of 3 points), chromatogram alignment (pairwise alignment based tree, RT search interval 200 scans, *m*/*z* window 5 points, RT window 5 scans, gap penalty 1), peak detection (summation window 0.09 min, minimum peak size 0.05 min, maximum merge distance 5 points, peak RT splitting enabled with maximum intensity profiling, gap/peak ratio 90%, smoothing window three points, curvature-based peak detection, peak refinement enabled at a refinement threshold of 80%, consistency filter at threshold 1). Positive and negative mode data were processed with the same settings, combined after export and further analyzed in R. The initial data matrix for LC-MS metabolites contained 249,893 features (characterized by *m*/*z* and RT information) and 301 samples (228 experimental samples, 57 control experiment samples, eight blanks, and eight nonfractionated samples). The data were then filtered as follows (excluding nonfractionated samples). To be further considered in analysis, a metabolic feature in any fractionated sample was required to be 10 times above the average blank intensity and to have a maximal intensity greater 5000. The resulting data were then normalized to the protein content determined for each fraction from which the sample was extracted. To reduce the feature list to potential protein-bound features, we filtered features to have in all four experimental replicates in at least three consecutive protein-containing fractions (A4 to C12) as deduced from protein measurements and the calibration curve. The resulting features (4381) were further filtered manually to remove noise features leaving 4229 features for further analysis.

### Data pre-processing: LC-MS metabolite annotation

All features were matched to an in-house library of authentic standards allowing retention time deviations of 0.05 min and *m*/*z* deviation of 0.002. In addition, a replicate extract of one experiment was analyzed by MetaSysX GmbH (MetaSysX GmbH, Potsdam, Germany) using identical chromatographic settings, and annotations were transferred to in-house measured data by estimating the RT shifts over the chromatogram following the most abundant feature in every 0.05-min window. The higher number of annotations reported here compared with those described in Ref. 11 results from the growth of our standard library, *e.g.* as a result of incorporation of 400 dipeptide standards. Granting the request of MetaSysX, RT information was removed from the supporting information but will be shared upon inquiry.

### Data pre-processing: LC-MS protein data

Raw data were analyzed using MaxQuant 1.5.2.8 (40) and its implemented search engine Andromeda (41) using the standard settings with minor changes: false discovery rate correction was set to 0.01, first search and MSMS search mass tolerance were set to 10 ppm; LFQ ratio count was set to 1; and variable modifications were set to Met oxidation, N-terminal acetylation, and Ser, Thr, and Tyr phosphorylation. As databases, we used the common contaminations database coming along with MaxQuant and the *Arabidopsis* proteome of canonical and isoform entries from Uniprot (http://www.uniprot.org/proteomes,^4^ UPID UP000006548, retrieved on March 17, 2017, containing 33,037 proteins, last modified on December 18, 2016). The data in the proteinGroups result table from 111 files were subsequently processed with R. First, we filtered out proteins that were contaminants and all decoy hits. Next, we required at least two unique peptides per protein group or at least one unique and one razor peptide per protein group if they made up more than 25% sequence coverage. This decreased the initially 7942 identified protein groups to 7214. We chose to use protein group raw intensity values as the type of quantitative data for subsequent analyses based on a comparison of the overall coefficient of variation distributions. We compared LFQ and raw intensities after different normalization scenarios and obtained the best results (lowest overall coefficient of variation) when using normalized raw intensity values. Therefore, for each sample we normalized raw intensity values to the sum of the 95 percentile values of all intensities and further to the maximum in each experimental replicate.

### Data pre-processing: Protein and metabolite profile peak deconvolution

To efficiently correlate peaks of proteins and metabolite features, we split the data profiles into single peaks by finding local valleys in the data. Therefore, we compared averaged profiles from biological replicates of proteins and metabolites, respectively, with their loess (locally weighted scatterplot smoothed) curve and filtered the detected valleys with specialized parameters as given below. Average profiles were calculated using the median of separately maximum normalized replicate experiments with missing values replaced by zero values. The parameters for the loess smoother were 0.17 span and window of 3. After that, we kept potential valleys as true if they met the following criteria: a peak width of at least three fractions from the last valley and a minimum drop in intensity of 30% relative to the previous peak for proteins and 50% for metabolites. We additionally required the potential peak to have a minimum of 5% base peak intensity for proteins and more than 10% base peak intensity and 750 intensity for metabolites. After splitting the profiles, we filtered the deconvoluted data for having at least two consecutive complete replicate groups, and for metabolite data in addition for having a minimal median intensity of 5000. Furthermore, we removed protein and metabolite feature peaks that eluted in the first protein fractions (< fraction A04) and metabolite features that eluted in the last two protein fractions (> fraction C08). This procedure resulted in a final number of 3325 metabolic feature peaks derived from 2830 unique features that we considered as being potentially protein-bound. The protein data consisted of 5527 peaks derived from 4627 single protein groups that were used for subsequent analyses.

### Correlation of profiles

Deconvoluted and median averaged profiles of metabolic features or annotated metabolites and proteins were correlated using Pearson correlation. This was done in R but can be easily performed in Excel using either PEARSON and CORREL function.

### Cloning and protein overexpression

Coding sequence (CDS) of *A. thaliana kphmt1* gene (AT2G46110) was cloned into the *E. coli* expression vector pET300 containing His_6_-tag at the N terminus of the Gateway cassette (Champion^TM^ pET Expression System, ThermoFisher Scientific) using specific primers (Table S7). The N-terminal 144 bp of a *kphmt1* CDS constituting mitochondrial signal peptide was deleted to increase protein solubility. CDS of *A. thaliana mti* gene (At2g05830) was cloned into the *E. coli* expression vector pET300 containing His_6_-tag at the N terminus of the Gateway cassette (Champion^TM^ pET Expression System, ThermoFisher Scientific) using specific primers (Table S7). BL21 Star^TM^ (DE3) *E. coli* cells (ThermoFisher Scientific) were used for protein overexpression. CDS of *gapc1/2*, *gapcp1,* and *gapcp2* were isolated from *Arabidopsis* cDNA and cloned into pENTR/D-TOPO (ThermoFisher Scientific) using specific primers (Table S7). It is important to mention that due to the high similarity between *gapc1* and *gapc2* (98%; cytosolic versions), it was not possible to isolate the single version, and for this reason it was designated *gapc1/2*. The *gapc1/2*, *gapcp1,* and *gapcp2* genes were cloned as a C-terminal fusion in the pDEST17 expression vector containing a His_6_-tag at the N terminus of the gateway cassette (Karlsruhe, Germany). Star^TM^ and Rosetta^TM^ cells expressing His_6_-*kphmt1*, His_6_-*mti*, His_6_-*gapc1/2*, His_6_-*gapcp1,* and His_6_-*gapcp2* were grown in Luria-Bertani (LB) broth containing the required antibiotics at 28 °C. Overnight culture was suspended 100 times in fresh media, grown to OD 0.4, induced by addition of 0.1 mm isopropyl 1-thio-β-d-galactopyranoside, and transferred to 16 °C for overnight incubation. Cells were harvested by centrifugation and disrupted with an EmulsiFlex C3 homogenizer (Avestin, Mannheim, Germany). MTI protein was purified using imidazole-gradient purification and nickel-nitrilotriacetic acid-agarose beads (Qiagen, Hilden, Germany). Protein purity was checked by SDS-PAGE. KPHMT1 bacterial lysate was used directly for the analysis. To obtain a highly pure protein, SEC was performed, and 15 fractions were collected. GAPC1/2, GAPCP1, and GAPCP2 were found in fractions A8–A10. Purity of the protein was confirmed by SDS-PAGE and Prometheus NT.48, and A8 fraction was then further used to perform protein thermal stability measurements. The human recombinant GAPDH protein was purchased from Sigma to perform further protein thermal stability measurements.

### Affinity purification assay

Custom Tyr–Asp–agarose beads were purchased from Cube Biotech (Monheim, Germany). Tyr–Asp was coupled using the amine (N′ beads) group of tyrosine or carboxylic (C′ beads) group or aspartic acid via an 11-carbon spacer. Beads were equilibrated (lysis buffer) before incubation with the lysate. Lysate was divided in three replicates (3 ml each) and incubated with 200 μl of agarose resin for 1 h on a rotating wheel at 4 °C (binding). Afterward, the lysate was transferred to a Mobicol “Classic” (35 μm pore size filter) column and washed with 10 ml of wash buffer (0.025 m Tris-HCl, pH 7.5, 0.5 m NaCl). The beads were incubated with 400 μl of 1 mm Tyr and 1 mm Asp for 1 h on a rotating wheel at 4 °C. The beads were incubated with 400 μl of 1 mm Tyr–Asp (Eurogentec, Belgium) for 1 h on a rotating wheel at 4 °C. Eluate was collected for the analysis. Proteins were precipitated with pre-cooled acetone (1:4) and further dried in a vacuum concentrator and stored at −20 °C. LC-MS/MS proteomics analysis was done as described above. Proteins reproducibly pulled with both N′ and C′ beads were assigned as putative Tyr–Asp interactors.

### Protein thermal stability measurements

GAPC1/2, GAPCP1, and GAPCP2 proteins were obtained as described above, and the human GAPDH recombinant protein was obtained from a commercial supplier (Sigma) and stored in 10 mm Tris buffer (pH 7.5, 0.5 m NaCl). Tyr–Asp (25 μm), 3PGA (250 μm), and other dipeptides (25 μm) were prepared in 1× PBS buffer for the measurements using GAPC1/2 protein. For GAPCP1 Tyr–Asp (25 μm), 3PGA (250 μm) and other dipeptides (25 μm) were prepared in 1× PBS buffer, whereas for GAPCP2 Tyr–Asp (50 μm), 3PGA (250 μm) and other dipeptides (50 μm) were prepared in 1× PBS buffer. In addition, for the human GAPDH Tyr–Asp (500 μm), 3PGA (500 μm) and other dipeptides (500 μm) were prepared in Tris buffer (10 mm Tris-HCl, pH 7.5, 0.5 m NaCl). In accordance, GAPDH (1 μm) and GAPCs (0.2–0.25 mg/ml) were diluted using Tris or PBS buffer, respectively. Capillaries were loaded into the Prometheus NT.48 (Nanotemper). Unfolding was detected during heating in a linear thermal ramp (2 °C min^−1^, 20–90 °C) with an excitation power of 60–100%. Temperature-dependent protein unfolding was determined from changes in tryptophan and tyrosine fluorescence at emission wavelengths of 350 and 330 nm. Melting temperatures were determined by detecting the maximum of the first derivative of the fluorescence ratios (*F*_350 nm_/*F*_330 nm_) as described previously (43).

### MST

MST measurements were performed using a Monolith NT.115 instrument (NanoTemper, München, Germany). Proteins (KPHMT1 and MTI) were labeled in phosphate buffer (PBS) using the Monolith^TM^ His-tag labeling kit RED-Tris-NTA kit (MO-L008) according to the user manual. PBS buffer was exchanged to Tris, pH 7.5 (binding buffer). Excitation was optimized by varying the LED power to yield emission intensities above 200 fluorescence arbitrary units, corresponding to 10–50 nm labeled protein. Monolith power was set to 40%. Premium coated capillaries were used to prevent sticking. His_6_-tag control peptide provided with the kit was used as a control. MO Affinity Analysis software was used to analyze (*K_d_* calculation) and visualize the data. Capillaries were loaded into the instrument assets of 13–16 point ligand titrations. d-Pantothenic acid hemicalcium salt (Sigma catalog no. 137-08–6) was dissolved in Tris, pH 7.5, buffer to 1 mm stock. For measurement, pantothenic acid was diluted to 500 μm working concentration. MTA (Sigma catalog no. D5011) was dissolved to 150 mm stock in DMSO and then diluted with Tris buffer, pH 7.5, to 1 mm working concentration.

### Data deposition

The MS proteomics data have been deposited to the ProteomeXchange Consortium via the PRIDE (42) partner repository with the dataset identifier. Metabolomics data were deposited into MetaboLights repository (37) as MTBLS94.

## Author contributions

D. V. performed the SEC experiment, analyzed the data, supervised the project, wrote the manuscript; E. M. S. performed validation experiments for pantothenate and methylthioadenosine, analyzed the data,wrote the manuscript; J. C. M. Tyr–Asp experiments; S. K. devised the concept of using SEC for separation of protein–small-molecule complexes; J. C. performed validation experiments for pantothenate and methylthioadenosine and contributed technical support; I. W., M. L., M. K., and J. S. contributed technical support; M. G. assistance with data deposition. M. M. was involved in the metabolomic analysis; A. G. and E. H. M. were involved in the proteomic measurements; L. W. devised the concept of using SEC for separation of protein–small-molecule complexes, supervised the project, and wrote the manuscript; A. S. analyzed the data, devised the concept of using SEC for separation of protein–small-molecule complexes, supervised the project, and wrote the manuscript.

## Supplementary Material

Supporting Information

## Footnotes

4 Please note that the JBC is not responsible for the long-term archiving and maintenance of this site or any other third party hosted site.

## Footnotes

3 The abbreviations used are:

PMI: protein–metabolite interaction
SEC: size-exclusion chromatography
CP: core particle
RP: regulatory particle
PLP: pyridoxal 5′-phosphate
PS: pantothenate synthetase
MTA: methylthioadenosine
MTR: 5′-methylthioribose
MTI: methylthioribose-1-phosphate isomerase
AP: affinity purification
GAPC: glyceraldehyde-3-P-dehydrogenase
3PGA: glyceraldehyde 3-phosphate
CDS: coding sequence
COR: correlation
MAX: maximum
ROC: receiver operating characteristic
MST: microscale thermophoresis
LFQ: label-free quantification
TPP: thermal proteome profiling
RT: retention time.

## References

[1] Li X., and Snyder M. (2011) Metabolites as global regulators: a new view of protein regulation. Bioessays 33, 485–489 10.1002/bies.201100026 21495048

[2] Yang G. X., Li X., and Snyder M. (2012) Investigating metabolite–protein interactions: an overview of available techniques. Methods 57, 459–466 10.1016/j.ymeth.2012.06.013 22750303 PMC3448827

[3] Tholl D., and Aharoni A. (2014) Small molecules: from structural diversity to signalling and regulatory roles. Plant J. 79, 541–543 10.1111/tpj.12635 25116909

[4] Li X., Gianoulis T. A., Yip K. Y., Gerstein M., and Snyder M. (2010) Extensive *in vivo* metabolite–protein interactions revealed by large-scale systematic analyses. Cell 143, 639–650 10.1016/j.cell.2010.09.048 21035178 PMC3005334

[5] Maeda K., Poletto M., Chiapparino A., and Gavin A. C. (2014) A generic protocol for the purification and characterization of water-soluble complexes of affinity-tagged proteins and lipids. Nat. Protoc. 9, 2256–2266 10.1038/nprot.2014.148 25167057

[6] Scholten A., Poh M. K., van Veen T. A., van Breukelen B., Vos M. A., and Heck A. J. (2006) Analysis of the cGMP/cAMP interactome using a chemical proteomics approach in mammalian heart tissue validates sphingosine kinase type 1-interacting protein as a genuine and highly abundant AKAP. J. Proteome Res. 5, 1435–1447 10.1021/pr0600529 16739995

[7] Lomenick B., Hao R., Jonai N., Chin R. M., Aghajan M., Warburton S., Wang J., Wu R. P., Gomez F., Loo J. A., Wohlschlegel J. A., Vondriska T. M., Pelletier J., Herschman H. R., Clardy J., Clarke C. F., and Huang J. (2009) Target identification using drug affinity responsive target stability (DARTS). Proc. Natl. Acad. Sci. U.S.A. 106, 21984–21989 10.1073/pnas.0910040106 19995983 PMC2789755

[8] Reinhard F. B., Eberhard D., Werner T., Franken H., Childs D., Doce C., Savitski M. F., Huber W., Bantscheff M., Savitski M. M., and Drewes G. (2015) Thermal proteome profiling monitors ligand interactions with cellular membrane proteins. Nat. Methods 12, 1129–1131 10.1038/nmeth.3652 26524241

[9] Huber K. V., Olek K. M., Müller A. C., Tan C. S., Bennett K. L., Colinge J., and Superti-Furga G. (2015) Proteome-wide drug and metabolite interaction mapping by thermal-stability profiling. Nat. Methods 12, 1055–1057 10.1038/nmeth.3590 26389571 PMC4629415

[10] Hulce J. J., Cognetta A. B., Niphakis M. J., Tully S. E., and Cravatt B. F. (2013) Proteome-wide mapping of cholesterol-interacting proteins in mammalian cells. Nat. Methods 10, 259–264 10.1038/nmeth.2368 23396283 PMC3601559

[11] Veyel D., Kierszniowska S., Kosmacz M., Sokolowska E. M., Michaelis A., Luzarowski M., Szlachetko J., Willmitzer L., and Skirycz A. (2017) System-wide detection of protein-small molecule complexes suggests extensive metabolite regulation in plants. Sci. Rep. 7, 42387 10.1038/srep42387 28205532 PMC5304321

[12] Aryal U. K., Xiong Y., McBride Z., Kihara D., Xie J., Hall M. C., and Szymanski D. B. (2014) A proteomic strategy for global analysis of plant protein complexes. Plant Cell 26, 3867–3882 10.1105/tpc.114.127563 25293756 PMC4247564

[13] Olinares P. D., Ponnala L., and van Wijk K. J. (2010) Megadalton complexes in the chloroplast stroma of *Arabidopsis thaliana* characterized by size exclusion chromatography, mass spectrometry, and hierarchical clustering. Mol. Cell. Proteomics 9, 1594–1615 10.1074/mcp.M000038-MCP201 20423899 PMC2938090

[14] Kristensen A. R., Gsponer J., and Foster L. J. (2012) A high-throughput approach for measuring temporal changes in the interactome. Nat. Methods 9, 907–909 10.1038/nmeth.2131 22863883 PMC3954081

[15] Hooper C. M., Castleden I. R., Tanz S. K., Aryamanesh N., and Millar A. H. (2017) SUBA4: the interactive data analysis centre for *Arabidopsis* subcellular protein locations. Nucleic Acids Res. 45, D1064–D1074 10.1093/nar/gkw1041 27899614 PMC5210537

[16] Tanahashi N., Murakami Y., Minami Y., Shimbara N., Hendil K. B., and Tanaka K. (2000) Hybrid proteasomes–induction by interferon-gamma and contribution to ATP-dependent proteolysis. J. Biol. Chem. 275, 14336–14345 10.1074/jbc.275.19.14336 10799514

[17] Finley D. (2009) Recognition and processing of ubiquitin–protein conjugates by the proteasome. Annu. Rev. Biochem. 78, 477–513 10.1146/annurev.biochem.78.081507.101607 19489727 PMC3431160

[18] Szklarczyk D., Santos A., von Mering C., Jensen L. J., Bork P., and Kuhn M. (2016) STITCH 5: augmenting protein–chemical interaction networks with tissue and affinity data. Nucleic Acids Res. 44, D380–D384 10.1093/nar/gkv1277 26590256 PMC4702904

[19] Han B. W., Malone T. E., Kim D. J., Bingman C. A., Kim H. J., Fox B. G., and Phillips G. N. (2011) Crystal structure of *Arabidopsis thaliana 12*-oxophytodienoate reductase isoform 3 in complex with 8-iso prostaglandin A(1). Proteins 79, 3236–3241 10.1002/prot.23153 21915915 PMC3192245

[20] Bonner E. R., Cahoon R. E., Knapke S. M., and Jez J. M. (2005) Molecular basis of cysteine biosynthesis in plants–structural and functional analysis of *O*-acetylserine sulfhydrylase from *Arabidopsis thaliana*. J. Biol. Chem. 280, 38803–38813 10.1074/jbc.M505313200 16166087

[21] Watanabe N., Cherney M. M., van Belkum M. J., Marcus S. L., Flegel M. D., Clay M. D., Deyholos M. K., Vederas J. C., and James M. N. (2007) Crystal structure of LL-diaminopimelate aminotransferase from *Arabidopsis thaliana*: a recently discovered enzyme in the biosynthesis of l-lysine by plants and *Chlamydia*. J. Mol. Biol. 371, 685–702 10.1016/j.jmb.2007.05.061 17583737

[22] Mooney S., and Hellmann H. (2010) Vitamin B6: killing two birds with one stone? Phytochemistry 71, 495–501 10.1016/j.phytochem.2009.12.015 20089286

[23] Myung J., Kim K. B., and Crews C. M. (2001) The ubiquitin–proteasome pathway and proteasome inhibitors. Med. Res. Rev. 21, 245–273 10.1002/med.1009 11410931 PMC2556558

[24] Ottenhof H. H., Ashurst J. L., Whitney H. M., Saldanha S. A., Schmitzberger F., Gweon H. S., Blundell T. L., Abell C., and Smith A. G. (2004) Organisation of the pantothenate (vitamin B5) biosynthesis pathway in higher plants. Plant J. 37, 61–72 10.1046/j.1365-313X.2003.01940.x 14675432

[25] Tilton G. B., Wedemeyer W. J., Browse J., and Ohlrogge J. (2006) Plant coenzyme A biosynthesis: characterization of two pantothenate kinases from *Arabidopsis*. Plant Mol. Biol. 61, 629–642 10.1007/s11103-006-0037-4 16897480

[26] Jerabek-Willemsen M., Wienken C. J., Braun D., Baaske P., and Duhr S. (2011) Molecular interaction studies using microscale thermophoresis. Assay Drug Dev. Technol. 9, 342–353 10.1089/adt.2011.0380 21812660 PMC3148787

[27] Powers S. G., and Snell E. E. (1976) Ketopantoate hydroxymethyltransferase. II. Physical, catalytic, and regulatory properties. J. Biol. Chem. 251, 3786–3793 6463

[28] Waduwara-Jayabahu I., Oppermann Y., Wirtz M., Hull Z. T., Schoor S., Plotnikov A. N., Hell R., Sauter M., and Moffatt B. A. (2012) Recycling of methylthioadenosine is essential for normal vascular development and reproduction in *Arabidopsis*. Plant Physiol. 158, 1728–1744 10.1104/pp.111.191072 22345506 PMC3320181

[29] Pommerrenig B., Feussner K., Zierer W., Rabinovych V., Klebl F., Feussner I., and Sauer N. (2011) Phloem-specific expression of Yang cycle genes and identification of novel Yang cycle enzymes in *Plantago* and *Arabidopsis*. Plant Cell 23, 1904–1919 10.1105/tpc.110.079657 21540433 PMC3123959

[30] Zierer W., Hajirezaei M. R., Eggert K., Sauer N., von Wirén N., and Pommerrenig B. (2016) Phloem-specific methionine recycling fuels polyamine biosynthesis in a sulfur-dependent manner and promotes flower and seed development. Plant Physiol. 170, 790–806 10.1104/pp.15.00786 26662272 PMC4734553

[31] Sauter M., Moffatt B., Saechao M. C., Hell R., and Wirtz M. (2013) Methionine salvage and *S*-adenosylmethionine: essential links between sulfur, ethylene and polyamine biosynthesis. Biochem. J. 451, 145–154 10.1042/BJ20121744 23535167

[32] Henderson B., and Martin A. C. (2014) Protein moonlighting: a new factor in biology and medicine. Biochem. Soc. Trans. 42, 1671–1678 10.1042/BST20140273 25399588

[33] Naka K., Jomen Y., Ishihara K., Kim J., Ishimoto T., Bae E. J., Mohney R. P., Stirdivant S. M., Oshima H., Oshima M., Kim D. W., Nakauchi H., Takihara Y., Kato Y., Ooshima A., and Kim S. J. (2015) Dipeptide species regulate p38MAPK-Smad3 signalling to maintain chronic myelogenous leukaemia stem cells. Nat. Commun. 6, 8039 10.1038/ncomms9039 26289811 PMC4560789

[34] Kosmacz M., Luzarowski M., Kerber O., Leniak E., Gutiérrez-Beltrán E., Moreno J. C., Gorka M., Szlachetko J., Veyel D., Graf A., and Skirycz A. (2018) Interaction of 2′,3′-cAMP with Rbp47b plays a role in stress granule formation. Plant Physiol. 177, 411–421 29618637 10.1104/pp.18.00285 PMC5933139

[35] Franken H., Mathieson T., Childs D., Sweetman G. M., Werner T., Tögel I., Doce C., Gade S., Bantscheff M., Drewes G., Reinhard F. B., Huber W., and Savitski M. M. (2015) Thermal proteome profiling for unbiased identification of direct and indirect drug targets using multiplexed quantitative mass spectrometry. Nat. Protoc. 10, 1567–1593 10.1038/nprot.2015.101 26379230

[36] Krueger S., Giavalisco P., Krall L., Steinhauser M. C., Büssis D., Usadel B., Flügge U. I., Fernie A. R., Willmitzer L., and Steinhauser D. (2011) A topological map of the compartmentalized *Arabidopsis thaliana* leaf metabolome. PLoS One 6, e17806 10.1371/journal.pone.0017806 21423574 PMC3058050

[37] Haug K., Salek R. M., Conesa P., Hastings J., de Matos P., Rijnbeek M., Mahendraker T., Williams M., Neumann S., Rocca-Serra P., Maguire E., González-Beltran A., Sansone S. A., Griffin J. L., and Steinbeck C. (2013) MetaboLights–an open-access general-purpose repository for metabolomics studies and associated meta-data. Nucleic Acids Res. 41, D781–D786 10.1093/nar/gks1004 23109552 PMC3531110

[38] Van Leene J., Eeckhout D., Cannoot B., De Winne N., Persiau G., Van De Slijke E., Vercruysse L., Dedecker M., Verkest A., Vandepoele K., Martens L., Witters E., Gevaert K., and De Jaeger G. (2015) An improved toolbox to unravel the plant cellular machinery by tandem affinity purification of *Arabidopsis* protein complexes. Nat. Protoc. 10, 169–187 25521792 10.1038/nprot.2014.199

[39] Giavalisco P., Li Y., Matthes A., Eckhardt A., Hubberten H. M., Hesse H., Segu S., Hummel J., Köhl K., and Willmitzer L. (2011) Elemental formula annotation of polar and lipophilic metabolites using (13)C, (15)N and (34)S isotope labelling, in combination with high-resolution mass spectrometry. Plant J. 68, 364–376 10.1111/j.1365-313X.2011.04682.x 21699588

[40] Cox J., and Mann M. (2008) MaxQuant enables high peptide identification rates, individualized p.p.b.-range mass accuracies and proteome-wide protein quantification. Nat. Biotechnol. 26, 1367–1372 10.1038/nbt.1511 19029910

[41] Cox J., Neuhauser N., Michalski A., Scheltema R. A., Olsen J. V., and Mann M. (2011) Andromeda: a peptide search engine integrated into the MaxQuant environment. J. Proteome Res. 10, 1794–1805 10.1021/pr101065j 21254760

[42] Vizcaíno J. A., Csordas A., Del-Toro N., Dianes J. A., Griss J., Lavidas I., Mayer G., Perez-Riverol Y., Reisinger F., Ternent T., Xu Q. W., Wang R., and Hermjakob H. (2016) 2016 update of the PRIDE database and its related tools. Nucleic Acids Res. 44, 11033 10.1093/nar/gkw880 27683222 PMC5159556

[43] Luzarowski M., Kosmacz M., Sokolowska E., Jasinska W., Willmitzer L., Veyel D., and Skirycz A. (2017) Affinity purification with metabolomic and proteomic analysis unravels diverse roles of nucleoside diphosphate kinases. J. Exp. Bot. 68, 3487–3499 10.1093/jxb/erx183 28586477 PMC5853561

