# Supplementary material for: PROMIS, global analysis of PROtein–metabolite interactions using size separation in Arabidopsis thaliana
Source: J Biol Chem. 2018 May 31;293(32):12440–53. doi: 10.1074/jbc.RA118.003351 (PMC6093232; doi:10.1074/jbc.RA118.003351)
Supplement: Supporting Information [file supp_293_32_12440__index.html]

PROMIS, global analysis of PROtein-Metabolite Interactions using Size separation in Arabidopsis thaliana — Global analysis of protein-metabolite interactions — PROMIS, global analysis of PROtein–metabolite interactions using size separation in Arabidopsis thaliana — Global analysis of protein–metabolite interactions — Supporting Information 

# PROMIS, global analysis of PROtein–metabolite interactions using size separation in *Arabidopsis thaliana*

## Supporting Information

- Datasets - Dataset S1-S8
- Figure S1 - Figure S1
- Tables - Tables S1-S7
