# Supplementary material for: PROMIS, global analysis of PROtein–metabolite interactions using size separation in Arabidopsis thaliana
Source: J Biol Chem. 2018 May 31;293(32):12440–53. doi: 10.1074/jbc.RA118.003351 (PMC6093232; doi:10.1074/jbc.RA118.003351)
Supplement: Supporting Information [file supp_RA118.003351_137284_1_supp_139212_p8tw1k.docx]

**Supporting Figures**

**
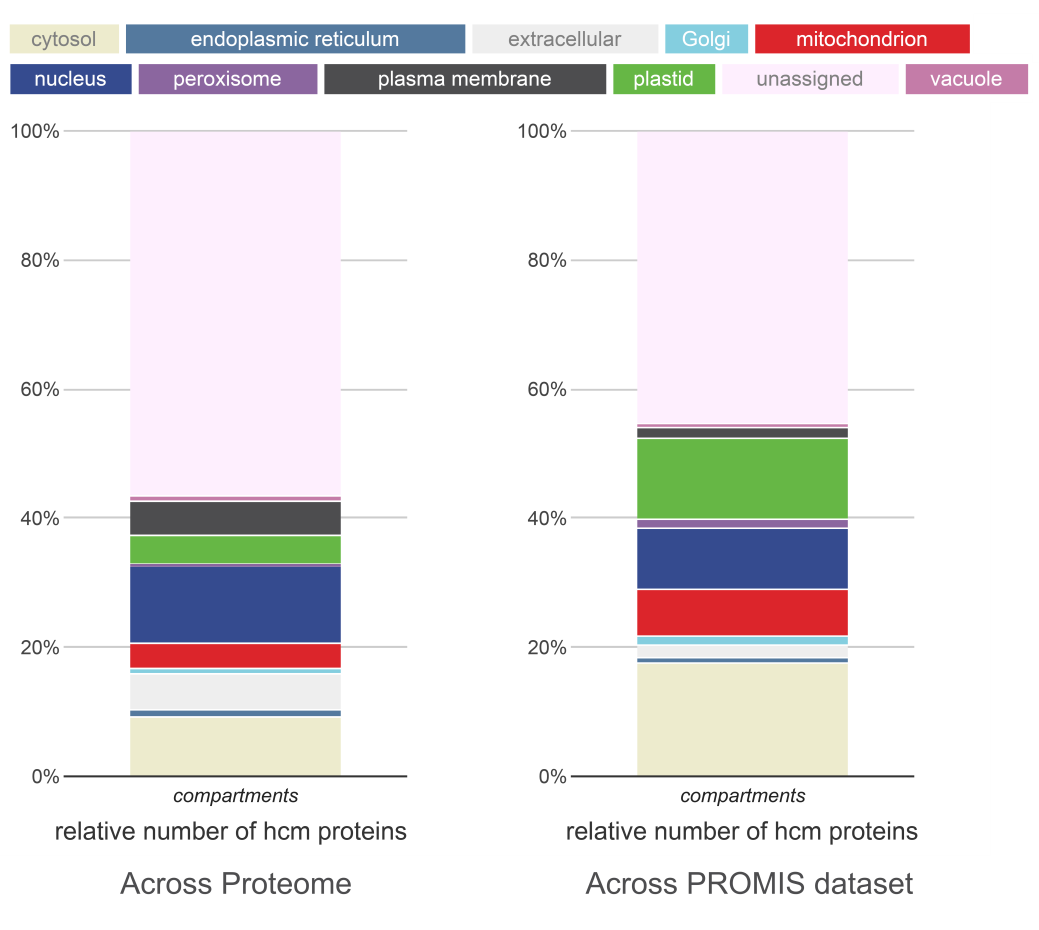
**

**Supporting Figure 1.** Subcellular distribution of the proteins present in our dataset in comparison to the subcellular distribution across the Arabidopsis proteome. Figure was generated using SUBA4 ([Hooper et al., 2017](#_ENREF_10)).
