# Supplementary material for: PROMIS, global analysis of PROtein–metabolite interactions using size separation in Arabidopsis thaliana
Source: J Biol Chem. 2018 May 31;293(32):12440–53. doi: 10.1074/jbc.RA118.003351 (PMC6093232; doi:10.1074/jbc.RA118.003351)
Supplement: Supporting Information [file supp_RA118.003351_137284_1_supp_139213_p8t71k.docx]

**Supporting Tables**

**Supporting Table S1**. List of the protein interactors retrieved from Stitch (>0.8 median confidence cut-off, experimental evidence for the Arabidopsis proteins) was used to query PROMIS dataset.

| **Uniprot** | **TAIR** | **Metabolite** | **Pearson correlation**  **(TRUE POSITIVES)*** | **Random 51 correlation (average n=10)**  **(FALSE POSITIVES)**** |
| --- | --- | --- | --- | --- |
| P25856 | At3g26650 | NAD | **0.97** | 0.84 |
| Q43725 | At3g59760 | Pyridoxal Phosphate | **0.94** | 0.79 |
| Q9C6D2 | At1g49820 | ADP | **0.94** | 0.73 |
| O48917 | At4g33030 | NAD | **0.93** | 0.70 |
| Q9MAT5 | At1g04870 | S-Adenosyl-L-homocysteine | **0.93** | 0.67 |
| O50008 | At5g17920 | Methionine | **0.93** | 0.62 |
| Q9SCL7 | At3g57560 | Arginine | **0.92** | 0.58 |
| Q9SUR9 | At4g23570 | ADP | **0.92** | 0.52 |
| Q0WP12 | At2g43910 | S-Adenosyl-L-homocysteine | **0.89** | 0.44 |
| Q9FUP0 | At2g06050 | FMN | **0.87** | 0.38 |
| Q94EG6 | At5g02240 | NADP | **0.85** | 0.33 |
| A0A1I9LQK6 | At3g57050 | Pyridoxal Phosphate | **0.83** | 0.27 |
| Q0PGJ6 | At2g37770 | NADP | **0.83** | 0.23 |
| Q93ZN9 | At4g33680 | Pyridoxal Phosphate | **0.82** | 0.17 |
| Q43125 | At4g08920 | FAD | **0.79** | 0.14 |
| Q8LGG8 | At3g01520 | AMP | **0.78** | 0.09 |
| Q96329 | At3g51840 | FAD | **0.77** | 0.06 |
| O80944 | At2g37760 | NADP | **0.74** | 0.02 |
| Q8LAH7 | At1g76680 | FMN | **0.74** | 0.00 |
| O04379 | At1g48410 | AMP | **0.74** | -0.04 |
| P47998 | At4g14880 | Pyridoxal Phosphate | **0.72** | -0.07 |
| Q9SD76 | At3g46970 | Pyridoxal Phosphate | **0.62** | -0.08 |
| O49482 | At4g34230 | NADP | **0.62** | -0.09 |
| B0F481 | At5g57590/At5g57600 | Pyridoxal Phosphate | **0.58** | -0.11 |
| P17597 | At3g48560 | FAD | **0.53** | -0.13 |
| Q39172 | At5g16970 | NADP | **0.52** | -0.14 |
| Q9C5D0 | At4g34120 | AMP | **0.48** | -0.15 |
| O65202 | At4g16760 | FAD | **0.46** | -0.16 |
| Q9C5Q8 | At4g20910 | S-Adenosyl-L-homocysteine | **0.42** | -0.16 |
| Q9SF23 | At3g11750 | Guanine | **0.33** | -0.18 |
| P69834 | At2g02500 | CMP | **0.30** | -0.19 |
| Q9FK51 | At5g18200 | AMP | **0.27** | -0.20 |
| Q9CAK8 | At1g63970 | CMP | **0.24** | -0.21 |
| Q9SWE5 | At3g18030 | FMN | **0.17** | -0.22 |
| Q9T0I8 | At4g38800 | S-Adenosyl-L-homocysteine | **0.09** | -0.22 |
| Q9SQT8 | At3g06350 | NADP | **0.05** | -0.22 |
| O04379 | At1g48410 | CMP | **0.02** | -0.23 |
| Q9SE33 | At5g51700 | ADP | **-0.09** | -0.23 |
| Q93YN9 | At5g42810 | ADP | **-0.11** | -0.23 |
| Q9FHN8 | At5g65930 | ADP | **-0.13** | -0.24 |
| Q9SCL7 | At3g57560 | ADP | **-0.14** | -0.24 |
| Q39054 | At5g20990 | AMP | **-0.14** | -0.25 |
| Q9LZP9 | At3g62410 | NAD | **-0.17** | -0.25 |
| Q96533 | At5g43940 | NAD | **-0.18** | -0.26 |
| Q93VR3 | At5g28840 | NAD | **-0.18** | -0.26 |
| Q9S7E4 | At5g14780 | NAD | **-0.19** | -0.27 |
| Q9ZST4 | At4g01900 | Arginine | **-0.19** | -0.28 |
| Q9ZST4 | At4g01900 | ADP | **-0.19** | -0.30 |
| Q39243 | At4g35460 | FAD | **-0.23** | -0.31 |
| Q84KJ5 | At5g24850 | FAD | **-0.25** | -0.32 |
| P46422 | At4g02520 | Glutamic Acid | **-0.33** | -0.34 |

*For the metabolites with multiple elution peaks we took the higher Pearson correlation. **A list of 51 random interactions was selected 10 times from the PROMIS dataset and used to calculate average.

**Supporting Table S2.** Lists of the protein interactors retrieved from Stitch (>0.4 median confidence cut-off, experimental evidence for the Arabidopsis homologs) were used to query PROMIS predicted PMIs interactions for the eight co-factors.

| **Co-factor** | **Peak Max** | **SEC (CORREL>0.7 and MAX +/- 1 fraction)** | **List from Stitch** | **Overlap** | **Enrichment** | ***p-value* (Fisher Exact Test)** |
| --- | --- | --- | --- | --- | --- | --- |
| Nicotinamide | 145kDa | 351 | 14 | 3 | **2.86508** | p <0.05 |
| Nicotinamide | 17kDa | 329 | 14 | 0 | ND | ND |
|  |  |  |  |  |  |  |
| Nicotinamide adenine dinucleotide | 506kDa | 430 | 187 | 10 | 0.58363 | p >0.05 |
| Nicotinamide adenine dinucleotide | 211kDa | 386 | 187 | 20 | 1.30032 | p >0.05 |
|  |  |  |  |  |  |  |
| Flavine adenine dinucleotide (FAD) | 164kDa | 367 | 153 | 13 | 1.08652 | p >0.05 |
|  |  |  |  |  |  |  |
| Flavine mononucleotide (FMN) | 113kDa | 355 | 86 | 4 | 0.61487 | p >0.05 |
| Flavine mononucleotide (FMN) | 42kDa | 502 | 86 | 10 | 1.08705 | p >0.05 |
|  |  |  |  |  |  |  |
| Nicotinamide adenine dinucleotide phosphate (NADP) | 574kDa | 379 | 165 | 3 | 0.22514 | p <0.05 |
| Nicotinamide adenine dinucleotide phosphate (NADP) | 186kDa | 427 | 165 | 9 | 0.59949 | p >0.05 |
| Nicotinamide adenine dinucleotide phosphate (NADP) | 88kDa | 386 | 165 | 22 | **1.62107** | p <0.05 |
| Nicotinamide adenine dinucleotide phosphate (NADP) | 25kDa | 353 | 165 | 3 | 0.24172 | p <0.05 |
|  |  |  |  |  |  |  |
| Pyridoxal phosphate | 211kDa | 355 | 70 | 11 | **2.07738** | p <0.05 |
| Pyridoxal phosphate | 68kDa | 414 | 70 | 20 | **3.23879** | p <0.05 |
|  |  |  |  |  |  |  |
| Riboflavin | 78kDa | 322 | 6 | 1 | 2.42909 | p >0.05 |
| Riboflavin | 47kDa | 438 | 6 | 1 | 1.78577 | p >0.05 |
|  |  |  |  |  |  |  |
| Adenosine 5'-phosphosulfate | 100kDa | 486 | 8 | 5 | **6.03524** | p <0.05 |

Enrichment and Fisher exact test were calculated using Easy Fisher Exact Test Calculator (http://www.socscistatistics.com)

**Supporting Table S3.** List of the PLP protein interactors retrieved from Stitch (>0.4 median confidence cut-off, experimental evidence) found in the PROMIS dataset.

|  |  | **211kDa** | **68kDa** |
| --- | --- | --- | --- |
|  |  | **Pyridoxal phosphate** | **Pyridoxal phosphate** |
| **Entry** | **Maximum** | **CORREL** | **CORREL** |
| B0F481 | 186kDa | 0.58 | -0.12 |
| F4HYF3 | 60kDa | -0.21 | 0.82 |
| F4I7I0 | 100kDa | -0.22 | 0.63 |
| O22765 | 29kDa | -0.23 | -0.20 |
| O23254 | 186kDa | 0.71 | -0.14 |
| O23254 | 88kDa | -0.27 | 0.77 |
| O23254 | 13kDa | -0.20 | -0.18 |
| O49543 | 186kDa | 0.79 | -0.15 |
| O49543 | 88kDa | -0.25 | 0.88 |
| O49543 | 22kDa | -0.24 | -0.21 |
| O49627 | 17kDa | -0.23 | -0.20 |
| O80448 | 394kDa | 0.23 | -0.19 |
| O80988 | 186kDa | 0.76 | -0.15 |
| P0DKC4 | 60kDa | -0.22 | 0.84 |
| P14671 | 447kDa | 0.15 | -0.14 |
| P14671 | 113kDa | -0.18 | 0.48 |
| P25269 | 113kDa | -0.15 | 0.44 |
| P42799 | 78kDa | -0.22 | 0.90 |
| P46248 | 78kDa | -0.25 | 0.92 |
| P46643 | 78kDa | -0.22 | 0.92 |
| P46644 | 68kDa | -0.24 | 0.92 |
| P46645 | 78kDa | -0.25 | 0.94 |
| P47998 | 60kDa | -0.20 | 0.72 |
| A0A1I9LQK6 | 186kDa | 0.83 | -0.17 |
| Q3E6S9 | 113kDa | -0.14 | 0.32 |
| Q42472 | 447kDa | 0.21 | -0.19 |
| Q42521 | 394kDa | 0.45 | -0.26 |
| Q42522 | 78kDa | -0.23 | 0.90 |
| Q42529 | 29kDa | -0.22 | -0.20 |
| Q43725 | 68kDa | -0.24 | 0.94 |
| Q56YA5 | 128kDa | 0.03 | 0.10 |
| Q6NPF4 | 53kDa | -0.19 | 0.33 |
| Q84W65 | 239kDa | 0.63 | -0.17 |
| Q84W65 | 78kDa | -0.24 | 0.95 |
| Q84WV0 | 239kDa | 0.77 | -0.20 |
| Q8GWU0 | 53kDa | -0.23 | 0.53 |
| Q8L493 | 53kDa | -0.21 | 0.59 |
| Q8L940 | 394kDa | 0.22 | -0.20 |
| A0A178VG19 | 394kDa | 0.28 | -0.17 |
| Q8W1X2 | 53kDa | -0.21 | 0.25 |
| Q93WX6 | 88kDa | -0.20 | 0.69 |
| Q93ZN9 | 88kDa | -0.23 | 0.82 |
| Q940M2 | 186kDa | 0.77 | -0.14 |
| Q949X7 | 88kDa | -0.23 | 0.86 |
| Q94A94 | 88kDa | -0.24 | 0.81 |
| Q94AL9 | 211kDa | 0.85 | -0.17 |
| Q94B78 | 186kDa | 0.79 | -0.15 |
| Q94C74 | 211kDa | 0.87 | -0.15 |
| Q94CE5 | 100kDa | -0.22 | 0.61 |
| Q94JQ3 | 211kDa | 0.87 | -0.17 |
| Q96255 | 78kDa | -0.24 | 0.91 |
| Q9FGS4 | 78kDa | -0.21 | 0.89 |
| Q9FNK4 | 88kDa | -0.22 | 0.84 |
| Q9FPH3 | 145kDa | 0.34 | -0.04 |
| Q9LDV4 | 100kDa | -0.24 | 0.69 |
| Q9LPM9 | 78kDa | -0.22 | 0.92 |
| Q9LR30 | 78kDa | -0.22 | 0.88 |
| Q9LTX3 | 113kDa | -0.14 | 0.25 |
| Q9LVY1 | 78kDa | -0.30 | 0.92 |
| A0A178VKW4 | 100kDa | -0.20 | 0.54 |
| B3H658 | 68kDa | -0.25 | 0.89 |
| Q9M8M7 | 88kDa | -0.22 | 0.87 |
| Q9M8M7 | 29kDa | -0.17 | -0.15 |
| Q9MA74 | 186kDa | 0.70 | -0.13 |
| Q9MAB6 | 17kDa | -0.21 | -0.19 |
| Q9S757 | 68kDa | -0.22 | 0.89 |
| Q9S7B5 | 113kDa | -0.18 | 0.46 |
| Q9S7E9 | 78kDa | -0.18 | 0.77 |
| Q9SD76 | 164kDa | 0.62 | -0.07 |
| Q9SHP0 | 78kDa | -0.23 | 0.93 |
| Q9SIE1 | 88kDa | -0.25 | 0.82 |
| Q9SIV0 | 145kDa | 0.35 | -0.06 |
| Q9SSP5 | 113kDa | -0.19 | 0.43 |
| Q9SZJ5 | 211kDa | 0.86 | -0.13 |
| Q9ZNR6 | 447kDa | 0.20 | -0.19 |
| Q9ZSS6 | 307kDa | 0.50 | -0.22 |
| Q9ZSS6 | 113kDa | -0.15 | 0.55 |
| Q94JS1 | 25kDa | -0.23 | -0.21 |
| Q6DBI9 | 25kDa | -0.21 | -0.19 |
| Q944L8 | 29kDa | -0.26 | -0.23 |
| Q9C969 | 53kDa | -0.21 | 0.51 |
| Q9CAP1 | 78kDa | -0.22 | 0.89 |
| Q9M169 | 78kDa | -0.19 | 0.80 |
| A8MRI5 | 88kDa | -0.25 | 0.90 |

**Supporting Table S4.** Overlap of proteins identified as putative Tyr-Asp interactors in the PROMIS and AP experiments.

| **Protein name** | **Uniprot** | **TAIR** | **Tyr-Asp (CORREL; PROMIS)** |
| --- | --- | --- | --- |
| Glyceraldehyde-3-phosphate dehydrogenase GAPC1 | P25858 | At3g04120 | 0.95 |
| Ankyrin repeat domain-containing protein 2B | Q29Q26 | At2g17390 | 0.94 |
| Enoyl-[acyl-carrier-protein] | Q9SLA8 | At2g05990 | 0.93 |
| Ubiquitin domain-containing protein DSK2a | Q9SII9 | At2g17190 | 0.92 |
| Ubiquitin domain-containing protein DSK2b | Q9SII8 | At2g17200 | 0.91 |
| Transketolase-2 | F4IW47 | At2g45290 | 0.89 |
| Mitochondrial import inner membrane translocase subunit TIM9 | Q9XGX9 | At3g46560 | 0.89 |
| Heat shock 70 kDa protein 6 | Q9STW6 | At4g24280 | 0.89 |
| Heat shock 70 kDa protein 9 | Q8GUM2 | At4g37910 | 0.89 |
| ATP synthase subunit alpha | P92549 | AtMg01190 | 0.88 |
| 10 kDa chaperonin | P34893 | At1g14980 | 0.87 |
| Ankyrin repeat domain-containing protein 2A | Q9SAR5 | At4g35450 | 0.86 |
| 20 kDa chaperonin | O65282 | At5g20720 | 0.86 |
| Heat shock 70 kDa protein 10 | Q9LDZ0 | At5g09590 | 0.82 |
| Chaperonin 60 subunit alpha 1 | P21238 | At2g28000 | 0.82 |
| Elongation factor 2 | Q9ASR1 | At1g56070/At1g56075 | 0.81 |
| Serine carboxypeptidase-like 49 | P32826 | At3g10410 | 0.79 |
| Mitochondrial import inner membrane translocase subunit TIM8 | Q9XGY4 | At5g50810 | 0.78 |
| ATP sulfurylase 1 | Q9LIK9 | At3g22890 | 0.72 |
| Elongation factor Tu | P17745 | At4g20360 | 0.71 |

**Supporting Table S5.** Glyceraldehyde-3-phosphate dehydrogenase‒Tyr-Asp binding assay using nanoDSF technology.

| **Experiment** | **Metabolite** | **GAPDH** | | | **GAPC1/2** | | | **GAPCP1** | | | **GAPCP2** | | |
| --- | --- | --- | --- | --- | --- | --- | --- | --- | --- | --- | --- | --- | --- |
| **#** | **Dipeptide** | **Tm (°C)** | **ΔTm** | **p-value** | **Tm (°C)** | **ΔTm** | **p-value** | **Tm (°C)** | **ΔTm** | **p-value** | **Tm (°C)** | **ΔTm** | **p-value** |
| 1 | none | 59.68 | 0 |  | 55.21 | 0 |  | 55.04 | 0 |  | 55.03 | 0 |  |
|  | TyrAsp | 61.9 | 2.2 | **0.0003** | 53.51 | -1.7 | **0.0002** | 53.48 | -1.56 | **0.022** | 53.43 | -1.6 | **0.0049** |
| 2 | none | 59.79 | 0 |  | 53.99 | 0 |  | 54.57 | 0 |  | 55.21 | 0 |  |
|  | TyrAsp | 61.10 | 1.31 | **0.00004** | 52.86 | -1.13 | **0.018** | 52.47 | -2.09 | **0.017** | 53.63 | -1.58 | **0.0034** |
| 3 | none | 60 | 0 |  | 55.09 | 0 |  | 55.76 | 0 |  | 55.49 | 0 |  |
|  | TyrAsp | 61.1 | 1.1 | **0.0047** | 53.98 | -1.11 | **0.00055** | 53.54 | -2.2 | **0.005** | 53.93 | -1.55 | **0.0034** |
| 4 | none | 60.01 | 0 |  | 53.99 | 0 |  | 55.06 | 0 |  | 54.87 | 0 |  |
|  | TyrAsp | 61.08 | 1.06 | **0.0006** | 52.86 | -1.13 | **0.018** | 53 | -2.06 | **0.00027** | 53.64 | -1.23 | **0.00001** |

Given is the average T_m_ separately for four independent experiments (n = 4; independent capillaries in one experiment). Negative T_m_ values represent destabilization of the protein, while positive values represent stabilization. A heteroscedastic two-tails student t-test (unequal variance) was performed to compare control (GAPC protein plus PSB buffer) and treatment (GAPC protein plus Tyr-Asp). P-value (≤ 0.05) and a shift in temperature of at least 1 °C were used to define binding.

**Supporting Table S6.** Glyceraldehyde-3-phosphate dehydrogenase dipeptides binding assay using nanoDSF technology.

| **Metabolite** | **GAPDH** | | | **GAPC1/2** | | | **GAPCP1** | | | **GAPCP2** | | |
| --- | --- | --- | --- | --- | --- | --- | --- | --- | --- | --- | --- | --- |
| **Dipeptide** | **Tm (°C)** | **ΔTm** | **p-value** | **Tm (°C)** | **ΔTm** | **p-value** | **Tm (°C)** | **ΔTm** | **p-value** | **Tm (°C)** | **ΔTm** | **p-value** |
| none | 60 | 0 |  | 53.99 |  |  | 55.06 | 0 |  | 54.87 | 0 |  |
| TyrAsp | 61.1 | **1.1** | **0.0047** | 52.85 | **-1.13** | **0.019** | 53 | -2.06 | **0.0002** | 53.64 | **-1.23** | **0.000018** |
| 3PGA | 62.8 | **2.8** | **0.000023** | 46.45 | **-7.53** | **0.0000023** | 56.78 | **1.71** | **0.0049** | 58.08 | **3.21** | **0.000017** |
| ProGlu | 60.1 | 0.1 | 0.67 | 54.23 | 0.24 | 0.008 | 54.96 | -0.1 | 0.0041 | 54.74 | -0.12 | 0.03 |
| HisTyr | 58.8 | **-1.2** | **0.006** | 54.35 | 0.36 | 0.07 | 55.04 | -0.02 | 0.883 | 55.09 | 0.22 | 0.39 |
| LeuPhe | 60.2 | 0.2 | 0.49 | 54.38 | 0.39 | 0.003 | 54.73 | -0.33 | 0.57 | 55.07 | 0.2 | 0.95 |
| ThrMet | 60.2 | 0.2 | 0.34 | 54.66 | 0.67 | 0.012 | 55.05 | -0.01 | 0.3 | 55.53 | 0.66 | 0.07 |
| TyrLeu | 62.2 | **2.2** | **0.00015** | 54.82 | 0.83 | 0.01 | 55.21 | 0.15 | 0.5 | 56.55 | 1.67 | 0.06 |
| GlyPro | 60.1 | 0.1 | 0.76 | 54.155 | 0.16 | 0.08 | 54.95 | -0.11 | 0.33 | 55.42 | 0.55 | 0.057 |

Given is the average T_m_ from one experiment (n = 4; independent capillaries). Negative T_m_ values represent destabilization of the protein, while positive values represent stabilization. A heteroscedastic two-tails student t-test (unequal variance) was performed to compare control (GAPC protein plus PSB buffer) and treatment (GAPC protein plus given dipeptide). P-value (≤ 0.05) and a shift in temperature of at least 1 °C were used to define binding

**Supporting Table S7.** Primers used in this work.

| **Primer** | **Sequence (5’-3’)** |
| --- | --- |
| KPHMT1 F | GGGGACAAGTTTGTACAAAAAAGCAGGCTTGCAAAAACACCGGAGAGGAGAAC |
| KPHMT1 R | GGGGACCACTTTGTACAAGAAAGCTGGGTACTTTGAAGGCTCCATGTTCTC |
| MIT F | GGGGACAAGTTTGTACAAAAAAGCAGGCTGCATGTCCGGCGAAGGAGAC |
| MIT R | GGGGACCACTTTGTACAAGAAAGCTGGGTATCAACGAGATGAGTTTCCTGTTATC |
| GAPC2 F | CACC ATGGCTGACAAGAAGATCAGA |
| GAPC2 R | TTAGGCCTTTGACATGTGAAC |
| GAPCP1 F | CACC ATGGCCTTCTCTTCTCTCCTC |
| GAPCP1 R | TTAGTGGCTGGCAGCTACCAA |
| GAPCP2 F | CACC **ATG**GCCTTATCTTCTCTCCTC |
| GAPCP2 R | TTAGCGGCTGGCTGCAACTAA |
